# Enhancement of the Therapeutic Capacity of Mesenchymal Stem Cells by Genetic Modification: A Systematic Review

**DOI:** 10.3389/fcell.2020.587776

**Published:** 2020-10-30

**Authors:** Jeanne Adiwinata Pawitan, Thuy Anh Bui, Wildan Mubarok, Radiana Dhewayani Antarianto, Retno Wahyu Nurhayati, Ismail Hadisoebroto Dilogo, Delvac Oceandy

**Affiliations:** ^1^Department of Histology, Faculty of Medicine, Universitas Indonesia, Jakarta, Indonesia; ^2^Stem Cell Medical Technology Integrated Service Unit, Dr. Cipto Mangunkusumo General Hospital, Faculty of Medicine, Universitas Indonesia, Jakarta, Indonesia; ^3^Stem Cell and Tissue Engineering Research Center, Indonesia Medical Education and Research Institute, Faculty of Medicine, Universitas Indonesia, Jakarta, Indonesia; ^4^Division of Cardiovascular Sciences, Faculty of Biology, Medicine and Health, Manchester Academic Health Science Centre, The University of Manchester, Manchester, United Kingdom; ^5^Division of Chemical Engineering, Department of Materials Engineering Science, Graduate School of Engineering Science, Osaka University, Toyonaka, Japan; ^6^Department of Biochemistry and Molecular Biology, Faculty of Medicine, Universitas Indonesia, Jakarta, Indonesia; ^7^Department of Orthopaedic and Traumatology, Dr. Cipto Mangunkusumo General Hospital, Faculty of Medicine, Universitas Indonesia, Jakarta, Indonesia; ^8^Department of Biomedical Science, Faculty of Medicine, Universitas Airlangga, Surabaya, Indonesia

**Keywords:** engineered MSCs, tumor, cancer, metastasis, bone defect, animal models

## Abstract

**Background:**

The therapeutic capacity of mesenchymal stem cells (also known as mesenchymal stromal cells/MSCs) depends on their ability to respond to the need of the damaged tissue by secreting beneficial paracrine factors. MSCs can be genetically engineered to express certain beneficial factors. The aim of this systematic review is to compile and analyze published scientific literatures that report the use of engineered MSCs for the treatment of various diseases/conditions, to discuss the mechanisms of action, and to assess the efficacy of engineered MSC treatment.

**Methods:**

We retrieved all published studies in PubMed/MEDLINE and Cochrane Library on July 27, 2019, without time restriction using the following keywords: “engineered MSC” and “therapy” or “manipulated MSC” and “therapy.” In addition, relevant articles that were found during full text search were added. We identified 85 articles that were reviewed in this paper.

**Results:**

Of the 85 articles reviewed, 51 studies reported the use of engineered MSCs to treat tumor/cancer/malignancy/metastasis, whereas the other 34 studies tested engineered MSCs in treating non-tumor conditions. Most of the studies reported the use of MSCs in animal models, with only one study reporting a trial in human subjects. Thirty nine studies showed that the expression of beneficial paracrine factors would significantly enhance the therapeutic effects of the MSCs, whereas thirty three studies showed moderate effects, and one study in humans reported no effect. The mechanisms of action for MSC-based cancer treatment include the expression of “suicide genes,” induction of tumor cell apoptosis, and delivery of cytokines to induce an immune response against cancer cells. In the context of the treatment of non-cancerous diseases, the mechanism described in the reviewed papers included the expression of angiogenic, osteogenic, and growth factors.

**Conclusion:**

The therapeutic capacity of MSCs can be enhanced by inducing the expression of certain paracrine factors by genetic modification. Genetically engineered MSCs have been used successfully in various animal models of diseases. However, the results should be interpreted cautiously because animal models might not perfectly represent real human diseases. Therefore, further studies are needed to explore the translational potential of genetically engineered MSCs.

## Introduction

Mesenchymal stem cells (MSCs) or mesenchymal stromal cells are adult multipotent stem cells that can be differentiated to other cell types such as bones, cartilage, skeletal muscles, and connective tissues. MSCs can be isolated from various sources, including bone marrow, adipose tissue, and the mucoid tissue within the umbilical cord (Wharton’s jelly) (Pawitan et al., 2013, 2014; Budiyanti et al., 2015). A large number of studies, including several double-blind randomized clinical trials, have reported the use of MSCs in treating various conditions (Mohamadnejad et al., 2013; Pawitan et al., 2018; Freitag et al., 2019). Most of the studies showed that MSCs exert their beneficial effects mainly through the secretion of paracrine factors (Lee et al., 2011; Mirotsou et al., 2011; Mohamadnejad et al., 2013; Freitag et al., 2019). Moreover, MSC-based therapy has obtained approval for clinical use in some countries. For example, MSC-based therapies have been approved for the treatment of acute graft-versus-host disease, critical limb ischemia (Buerger’s disease), and complex perianal fistulas in adults due to Crohn’s disease in Japan, India, and the European Union, respectively (Robb et al., 2019). It is thought that the therapeutic capacity of MSCs largely depends on their ability to secrete beneficial factors to repair the damaged tissues or organs. Therefore, genetically engineered MSCs that can specifically express paracrine factors, which are needed in a particular pathological condition, may be more effective in treating the disease compared to native MSCs. The aim of this article is to systematically review the literatures on the use of genetically engineered MSCs in various diseases and to assess their safety and efficacy. This review will provide an update on the mechanisms of action of engineered MSCs in the treatment of various conditions.

## Methods

This systematic review complies with the Preferred Reporting Items for Systematic Review (PRISMA) guidelines. The protocol was submitted to the PROSPERO database of systematic review protocols (registration number: CRD 42020163707). We have checked the Cochrane Library to ensure that there was no systematic review on a similar topic.

We performed a PubMed/MEDLINE and Cochrane database search for relevant published studies on July 27, 2019, without time restriction using the following search terms: “engineered MSC” AND “therapy” or “manipulated MSC” AND “therapy.” In addition, relevant articles found during full text search were reviewed.

All original articles on the therapeutic use of engineered MSC or manipulated MSC were included. Studies on engineered MSC or manipulated MSC therapy that did not provide data on the treated conditions or disease models, type of MSC manipulation, detail information on expressed protein, administration route, and outcome of the treatment were excluded from this review. Article selection was conducted by examination of the titles, abstracts, and full texts.

The data collected include the following: the treated disease or disease models, the species used, the number of animals/participants, the source of the MSC, MSC passage, the type of gene insertion and expressed protein, the outcomes of the treatment, adverse events, and mechanism of action.

Data analysis: the studies were grouped and tabulated according to the treated conditions/disease models, species, number of animals, MSC source and passage, vector used, types of gene insertion/expressed proteins, outcome, adverse event, and mechanism of action.

Data were analyzed descriptively to determine the safety and efficacy of a certain engineered MSC on a certain disease and the possible mechanism.

## Results and Discussion

**FIGURE 1:**
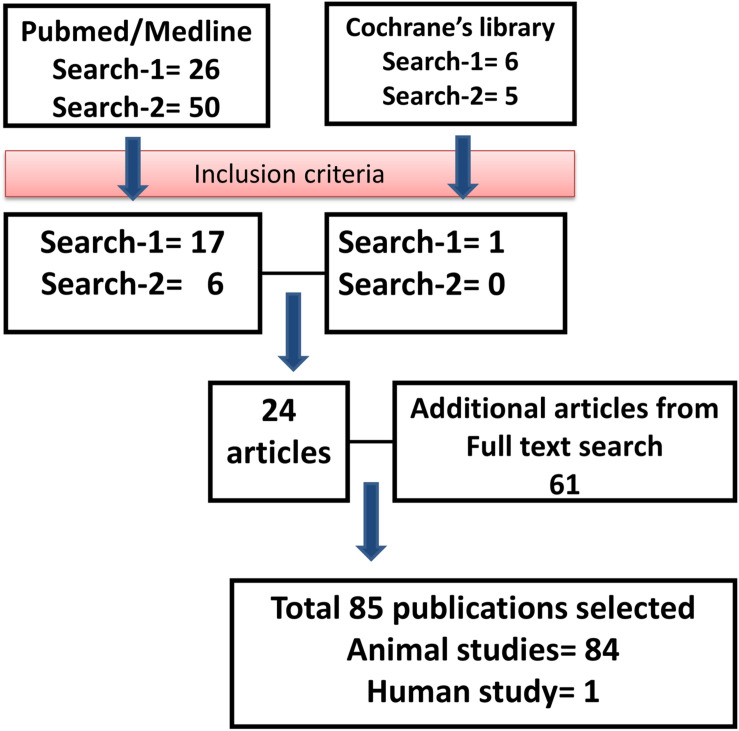
Outline of the literature search. Flowchart describing the protocol and literature search used in this systematic review.

Using the keywords “engineered MSC” AND “therapy,” we identified 26 articles from the PubMed database, of which 17 articles were included for review. Six articles from the Cochrane Library database were identified using the same keywords (of which one was selected for review). Using the keywords “manipulated MSC” AND “therapy,” we identified 50 articles from the PubMed database, of which six papers were selected for review. The same keywords were used in the Cochrane database, and we have identified five articles, but none of them met the inclusion criteria for review. During full text search, we found 61 relevant articles that met the inclusion criteria, and thus, the total number of articles included in the review was 85 (Figure 1).

The conditions/disease models treated by engineered MSC were grouped into two main groups: (i) tumor, cancer, malignancy, and metastasis (Supplementary Table 1) and (ii) non-tumor conditions (Supplementary Table 2).

Engineered MSCs were mostly used to treat tumor/cancer/malignancy/metastasis (51 studies, Supplementary Table 1), and the rest of the studies reported the use of engineered MSCs to treat various non-tumor conditions (34 studies, Supplementary Table 2). MSCs from several different sources and species were used in the reviewed papers, i.e., human bone marrow (BM) MSCs (Sasportas et al., 2009; You et al., 2009; Chang et al., 2010; Gao et al., 2010; Wang et al., 2012; Altaner et al., 2014; Harati et al., 2015; NguyenThai et al., 2015; Nouri et al., 2015; Beegle et al., 2016; Chung et al., 2016; Schug et al., 2018; Segaliny et al., 2019), human adipose tissue (AT) MSCs (Kucerova et al., 2007, 2008, 2014; Cavarretta et al., 2010; Altanerova et al., 2012; Zolochevska et al., 2012; Altaner et al., 2014; Grisendi et al., 2015; Matuskova et al., 2015; Tyciakova et al., 2015; Toro et al., 2016; Roudkenar et al., 2018), rat BM-MSCs (Tsuchida et al., 2003; Li et al., 2007; Jiang et al., 2009; Huang et al., 2010; Zhang et al., 2010, 2011; Zhao et al., 2010; Fei et al., 2012; Kosaka et al., 2012; Kim et al., 2013; Nakamura et al., 2013; Hu et al., 2014; Niu et al., 2014; Qi et al., 2015; Yao et al., 2017; Roudkenar et al., 2018), human embryonic stem cell (ESC) derived MSC (Bak et al., 2011), murine BM-MSCs (Kumar and Ponnazhagan, 2007; Chen et al., 2008; Ren et al., 2008; Xin et al., 2009; Conrad et al., 2011; Zhu et al., 2012; Zou et al., 2012; Fransson et al., 2014; Amara et al., 2016), mouse AT-MSC (Abrate et al., 2014; Krasikova et al., 2015, Krassikova et al., 2016), canine AT-MSCs (Seo et al., 2011; Ahn et al., 2013), human umbilical cord (UC) MSCs (Yan et al., 2013), rabbit BM-MSCs (Chang et al., 2004; Lin et al., 2010, 2012a; Fu et al., 2015; Wang et al., 2015b), pig BM-MSCs (autologous) (Chang et al., 2003; Chang et al., 2010), pig AT-MSCs (Lin et al., 2015), and rabbit AT-MSCs (Lin et al., 2011, 2012b; Lin et al., 2013). Only 2 out of 85 studies used autologous cells (Chang et al., 2003; Chang et al., 2010), and most of the reported studies used allogeneic cells. Some studies reported the use of human-derived MSCs in animal models, which could be regarded as xenogeneic cells, but no adverse immune reaction was reported. This might be due to the immune privilege and immune modulation properties of MSCs (Andreeva et al., 2017).

The genetic modification of the MSCs was achieved using different approaches, including viral and non-viral approaches. The types of viral vectors used for MSC genetic manipulation include the following: adenovirus (Chang et al., 2003, 2004; Chang et al., 2010; Tsuchida et al., 2003; Chen et al., 2008; Fritz et al., 2008; Jiang et al., 2009; Xin et al., 2009; Gao et al., 2010; Huang et al., 2010; Zhang et al., 2010; Zhao et al., 2010; Kosaka et al., 2012; Wang et al., 2012; Kim et al., 2013), lentivirus (Sasportas et al., 2009; Luetzkendorf et al., 2010; Bak et al., 2011; Seo et al., 2011; Zhang et al., 2011; Fei et al., 2012; Zolochevska et al., 2012; Ahn et al., 2013; Lee et al., 2013; Martinez-Quintanilla et al., 2013; Yan et al., 2013, 2017; Fransson et al., 2014; Niu et al., 2014; Qi et al., 2015; Wang et al., 2015b; Amara et al., 2016; Beegle et al., 2016), retrovirus (Kucerova et al., 2007, 2008, 2014; Cavarretta et al., 2010; Chang et al., 2010; Altanerova et al., 2012; Zou et al., 2012; Martinez-Quintanilla et al., 2013; Abrate et al., 2014; Grisendi et al., 2015; Lakota et al., 2015; Matuskova et al., 2015; Tyciakova et al., 2015; Chung et al., 2016; Toro et al., 2016), baculovirus (Lin et al., 2010, 2011, 2012a,b; Lin et al., 2015; Bak et al., 2011; Fu et al., 2015), adeno associated virus (Kumar and Ponnazhagan, 2007; Ren et al., 2008), and cytomegalovirus (Conrad et al., 2011). The non-viral approaches reported include the following: mRNA–lipofectamine-mediated transfection (Segaliny et al., 2019), PEGylated DNA-templated nano-composite system (Suryaprakash et al., 2019), plasmid DNA transfection (You et al., 2009; Harati et al., 2015; Krasikova et al., 2015, Krassikova et al., 2016; NguyenThai et al., 2015; Nouri et al., 2015; Mirzaei et al., 2018; Roudkenar et al., 2018; Schug et al., 2018), biotinylated MSCs (avidin link protein) (Yao et al., 2017), spermin pullulan (Nakamura et al., 2013; Hu et al., 2014), hyper-branched poly-amido-amine (hPAMAM) (Zhu et al., 2012), and jetPEI mediated transfection (Li et al., 2007). Most of the approaches were successful in transferring various genes into the MSCs and in inducing secretion of the protein of interest.

It is important to note that in gene therapy experiments, viral vectors – particularly adenovirus – often cause a strong immune reaction against the vector (Xin et al., 2009). However, when used to transfect MSCs *in vitro*, the transfected MSCs did not induce an adverse immune reaction following transplantation to host tissues (Xin et al., 2009).

### The Use of Engineered MSCs to Treat Tumor/Cancer/Malignancy/Metastasis

Despite recent improvements in prognosis and treatment modalities, cancer remains one of the major causes of death, particularly when metastasized tumors become resistant to conventional surgical and chemoradiotherapeutic treatment strategies (Chulpanova et al., 2018). However, recent studies on stem cell-based therapies have provided promising results in the development of new strategies for cancer treatment (Zhang et al., 2017). MSCs appear to be one potential candidate for stem cell-based anti-cancer therapies. MSCs are a group of adult stem cells that exert tumor tropism behavior and play various roles in regulating cancer cell biology. Therefore, these cells have been used as vehicles to deliver substances to the primary tumor or metastatic tumor sites.

Mesenchymal stromal cells have attracted considerable attention in the field of tumor therapy because of their unique biological properties. In most circumstances, tumor cells reside in a complex microenvironment, which also contains other cell types such as endothelial cells, fibroblasts, and other bone marrow-derived cells such as macrophages (Lin et al., 2019). Within this microenvironment complex, tumor cells secret a spectrum of chemokines, cytokines, and growth factors to attract MSCs to the tumor sites. Meanwhile, MSCs could secrete cytokines or chemokines, which orchestrate the fate and development of tumor cells. MSCs are also safe to be used in allogeneic transplantation because they do not express co-stimulatory molecules that trigger graft rejection and immune responses (Chulpanova et al., 2018).

Some pre-clinical studies have shown that MSCs could be genetically manipulated to express cytotoxic agents to inhibit tumor growth (Lin et al., 2019). There are various kinds of studies using different strategies and animal models to test the efficacy of engineered MSCs for the treatment of cancer/metastasis. In some studies, engineered MSCs were delivered after the tumors or metastatic lesions were established in animal models (Chen et al., 2008; Ren et al., 2008; Xin et al., 2009; Gao et al., 2010; Bak et al., 2011; Conrad et al., 2011; Zhang et al., 2011; Wang et al., 2012; Kim et al., 2013; Lee et al., 2013; Martinez-Quintanilla et al., 2013; Yan et al., 2013; Abrate et al., 2014; Altaner et al., 2014; Hu et al., 2014; Harati et al., 2015; Krasikova et al., 2015, Krassikova et al., 2016; Lakota et al., 2015; Matuskova et al., 2015; Nouri et al., 2015; Chung et al., 2016; Yan et al., 2017; Yao et al., 2017; Mirzaei et al., 2018; Schug et al., 2018; Segaliny et al., 2019; Suryaprakash et al., 2019). In other studies, tumor cells and engineered MSCs were injected simultaneously in animal models (Fritz et al., 2008; Sasportas et al., 2009; You et al., 2009; Cavarretta et al., 2010; Chang et al., 2010; Seo et al., 2011; Altanerova et al., 2012; Fei et al., 2012; Zolochevska et al., 2012; Ahn et al., 2013; Kucerova et al., 2014; Grisendi et al., 2015; NguyenThai et al., 2015; Tyciakova et al., 2015), whereas other studies used the combination of both approaches (Kucerova et al., 2007, 2008; Luetzkendorf et al., 2010; Kosaka et al., 2012; Zou et al., 2012; Amara et al., 2016; Toro et al., 2016). Co-injection of a mixture of tumor cells and engineered MSCs was intended to know whether engineered MSCs could prevent tumor development. However, this approach is not suitable in the clinical setting because patients are treated when the tumors are already established. The use of engineered MSCs for cancer prevention may not be feasible for clinical application because we do not know which patient will develop a certain type of tumor.

The genetically engineered MSCs are often used in conjunction with treatment with chemotherapeutic drugs. Thus, it is crucial to understand the effects of the chemotherapeutic agents on the MSCs themselves. MSCs’ responses to chemotherapeutic drugs vary depending on the mechanisms of action of the anti-cancer drugs used. However, in general, MSCs can be considered as more resistant cells against chemotherapeutic agents because of their highly efficient DNA repair, higher level of anti-apoptotic protein, and higher anti-oxidant activity as reviewed in previous publications (Ruhle et al., 2018, 2019).

**FIGURE 2:**
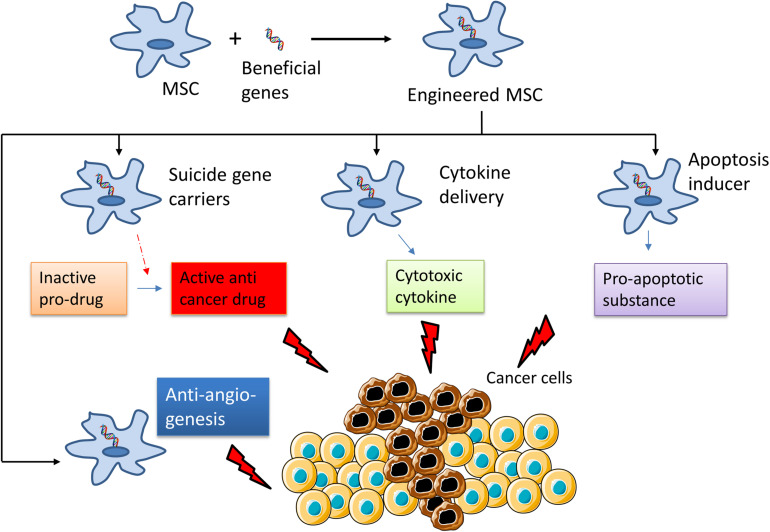
Summary of the mechanisms of the use of engineered Mesenchymal Stem Cells for cancer treatment. MSCs are genetically modified to express “suicide genes,” which can activate an inactive pro-drug to active anti-cancer drug; cytotoxic cytokines or apoptosis inducer that can kill cancer cells. Some part of this figure was created using Servier Medical Art templates, which are licensed under a Creative Commons Attribution 3.0 Unported License; https://smart.servier.com.

There are three different strategies to generate anti-cancer MSCs through genetic modification: (i) by inserting suicide gene carriers that will activate a non-toxic pro-drug to become a cytotoxic substance that can kill the tumor cells, (ii) by using MSCs as vehicles to deliver cytokines for cancer immunotherapy; and (iii) by using MSCs as agents to induce tumor cell apoptosis (Figure 2).

#### MSCs as Suicide Gene Carriers That Activate Non-toxic Pro-drugs Into Toxic Substances

One of the main challenges of the current first-line cancer therapies, especially chemotherapy, is systemic cytotoxicity, and limited delivery to the tumor sites. To tackle these problems, genetically modified MSCs carrying suicide genes have been employed to convert non-toxic pro-drugs into active agents for selective elimination of cancer cells. Various genes encoding these “suicide proteins” were used: cytosine deaminase (CD) or cytosine deaminase-uracil phosphoribosyltransferase (CD-UPRT), which converts the pro-drug 5-fluorocytosine (5-FC) into an active agent 5-fluorouracil (5-FU) (Kucerova et al., 2007, 2014; You et al., 2009; Cavarretta et al., 2010; Chang et al., 2010; Conrad et al., 2011; Altanerova et al., 2012; Fei et al., 2012; Kosaka et al., 2012; Abrate et al., 2014; Krasikova et al., 2015, Krassikova et al., 2016; Lakota et al., 2015; Matuskova et al., 2015; NguyenThai et al., 2015; Chung et al., 2016; Toro et al., 2016; Segaliny et al., 2019); herpes simplex virus thymidilate kinase (HSV-TK) (Bak et al., 2011; Martinez-Quintanilla et al., 2013; Nouri et al., 2015) or SV40-TK (Lee et al., 2013), which phosphorylates gancyclovir (GCV) into a toxic substance; and cytochrome P450 reductase (CYP), which converts cyclophosphamide (CPA) into cytotoxic metabolites (Amara et al., 2016).

A study showed that human AT-MSCs expressing cytosine deaminase (CD) could trigger dose-dependent apoptosis of human melanoma A375 cells in both *in vitro* and *in vivo* models (Kucerova et al., 2008). Subcutaneous injection of 20% AT-MSC-CD in a mixture with A375 cells resulted in a complete regression of 89% of the tumor-bearing animals within 14 days (Kucerova et al., 2008). Moreover, AT-MSC-CD administered systemically exhibited tumor tropism and suppressed tumor growth in the presence of 5-FC.

Other pieces of evidence showing the promising therapeutic potential of MSCs in suppressing melanoma tumors have been provided by a study using a more aggressive variant of melanoma cells, that is, EGFP-A375/Rel3, which exhibited altered cell adhesion and tumorigenic and metastatic properties (Kucerova et al., 2014). The combination of AT-MSC-CD/5-FC treatment with SU11274, an inhibitor of the c-Met/hepatocyte growth factor signaling pathway, could provide a complete cure in 9 out of 10 animals at 60 days after EGFP-A375/Rel3 cell injection (Kucerova et al., 2014). Augmentation of CD with herpes virus 1 (HSV-1) tegument protein VP22 in a CD-UPRT fusion construct could also enhance the therapeutic effects of the MSC-CD-UPRT combination (Krasikova et al., 2015). Consistent with studies using animal models, human BM-MSC-CD could migrate to the subcutaneous human gastric cancer MKN45 cells and induce tumor regression in the presence of 5-FC (You et al., 2009). Importantly, these studies have suggested the importance of identifying optimal timing and augmentation of the treatment to maximize the anti-tumor effects of the MSCs.

As mentioned previously, one major issue on the use of MSCs in combination with chemotherapeutic substances is the toxicity of the chemotherapeutic drugs on the MSCs. MSCs have been shown to have some degree of resistance against alkylating agents such as cyclophosphamide, melphalan, and busulfan (Nifontova et al., 2008; Kemp et al., 2011). It has also been reported that MSCs displayed high resistance against cisplatin, a platinum-based anti-cancer drug (Bellagamba et al., 2016; Nicolay et al., 2016). In addition, treatment with methotrexate did not affect the survival and proliferative capacity of MSCs (Mancheno-Corvo et al., 2013; Beane et al., 2014). However, studies on the effects of nucleoside analog 5-FU on MSCs are rather limited. One study suggested that low doses of 5-FU reduced MSC viability. However, others have demonstrated a lower level of senescence in MSCs treated with 5-FU compared to those treated with other chemotherapeutics agents such as doxorubycin, methotrexate, or busulfan (Qi et al., 2012). The origin of the MSCs may also determine that the response to 5-FU with adipose-derived MSCs appears to have lower sensitivity against 5-FU compared to bone marrow-derived MSCs (Ruhle et al., 2018).

#### MSCs as Vehicles for Cytokine Delivery in Cancer Immunotherapy

In addition to the application in delivering “suicide genes,” engineered MSCs have been used as tools to deliver anti-cancer cytokines to the tumor local environment. Thus, treatment using engineered MSCs has been regarded as one important approach in the field of cancer immunotherapy In a study on 786-0 renal cancer cell xenografts, MSCs expressing interleukin-12 could migrate to the tumor site and inhibit tumor growth through the activation of the natural killer cells and secretion of interferon γ at 14 days after MSC administration (Gao et al., 2010). In a mouse model of subcutaneous B16F10 melanoma xenograft, a combined treatment of canine AT-MSCs expressing interferon β (IFN-β) along with systemic administration of the platinum-containing anti-cancer drug cisplatin could significantly inhibit tumor growth at 14 days after tumor cell injection compared with a cisplatin monotherapy group. This method could also increase mouse survival up to 51 days after tumor cell injection compared to 20 days of survival in the control group (Seo et al., 2011). Consistently, subcutaneous injection of AT-MSC-IFN-β along with a low dose of systemic cisplatin could induce cell cycle arrest and apoptosis of LMeC melanoma xenografts (Ahn et al., 2013).

Another study reported that engineered MSCs expressing inflammatory cytokine TNFα could trigger caspase 3/7-dependent apoptosis of human A375 melanoma cells *in vitro* (Tyciakova et al., 2015). This study also showed that the subcutaneous injection of MSC-hTNFα and A375 melanoma cells in a 1:4 ratio could induce tumor regression up to 97.5% in the recipient mice.

In addition to the anti-tumor effects at the primary tumor site, the engineered MSCs might be able to control metastasized tumor growth as demonstrated in several studies. For example, systemic administration of hAT-MSC-CXCL10 could inhibit Treg cell-driven lung metastasized tumors and elevate the number of anti-tumor activated T-cells in the lungs in animals receiving B16F10-induced melanoma cells (Mirzaei et al., 2018). On the other hand, systemic administration of MSC–lipocalin 2 could inhibit liver metastasis through the downregulation of vascular endothelial growth factors in the liver in a mouse model of SW48 colon cancer intrasplenic xenografts (Harati et al., 2015). Together, these studies suggested that cytokine-based targeted therapy using MSCs might be effective in tackling a range of tumor cell types and possibly cancer metastasis.

#### Engineered MSCs That Target Tumor Cell Apoptosis

Another strategy in using MSCs for cancer therapy is to use these cells to deliver pro-apoptotic proteins to tumor cells (Sasportas et al., 2009; Luetzkendorf et al., 2010; Kim et al., 2013; Yan et al., 2013; Grisendi et al., 2015; Suryaprakash et al., 2019). Tumor necrosis factor (TNF)-related apoptosis-inducing ligand (TRAIL) is a transmembrane protein that binds to death domain-containing receptors and selectively triggers the extrinsic apoptotic pathway within cancer cells while sparing other cell types (Luetzkendorf et al., 2010). Because most current chemotherapy agents mainly act as DNA damage sensors and activate the intrinsic apoptotic pathway of cancer cells, MSC-TRAIL could be used in conjunction with first-line clinical therapies to provide synergistic treatment effects.

The therapeutic potential of MSC-TRAIL has been demonstrated in several pre-clinical models of sarcoma, lung cancer metastasis, renal cancer, colorectal cancer, and lymphomas. For example, co-injection of mixtures of human MSC-TRAIL with mouse colorectal cancer cells could effectively inhibit tumor growth via apoptosis activation. However, this therapeutic effect was abolished when TRAIL-MSCs were delivered through intravenous injection (Luetzkendorf et al., 2010). MSCs expressing human TRAIL could induce caspase 8 activation and apoptosis of RD-ES sarcoma cells while retaining anti-angiogenic effects (Grisendi et al., 2015). Engineered MSCs expressing TRAIL and herpes simplex virus thymidine kinase (MSC-TRAIL-TK) have shown efficacy in inducing apoptosis of renal carcinoma (RENCA) cells and inhibiting lung metastasis. Triple intravenous injections of MSC-TRAIL-TK could offer 100% survival rate and induce a complete cure for metastatic tumor-bearing mice (Kim et al., 2013).

mesenchymal stromal cell-TRAIL can be modified further to enhance their binding specificity to tumor cells. In a study using a mouse model of non-Hodgkin’s lymphoma, engineered MSCs expressing both CD20-specific single-chain Fv antibody fragment and TRAIL could migrate to established CD20-positive B-cell lymphoma line (BJAB) subcutaneous tumor in NOD/SCID mice and induce 65% tumor regression without causing liver toxicity compared to 42.7% of tumor regression in groups receiving MSC-TRAIL only (Yan et al., 2013).

So far, most studies using pre-clinical cancer models have shown consistent results, i.e., the engineered MSCs could inhibit tumor growth and enhance the survival rate of the tumor-bearing animals. However, a recent clinical report of a patient treated with engineered MSC-CD-UPRT and 5-FC for metastatic head and neck cancer has shown that there was no sign of tumor regression at 6 days after intravenous injection of the therapeutic stem cells. Moreover, tumor metastasis further progressed after 40 days of the treatment (Lakota et al., 2015). Therefore, success in animal studies should be interpreted with caution. Further studies to understand the biology, fate, and safety of grafted MSCs would be needed for successful clinical translation of MSC-based cancer therapies.

#### Other Genetic Strategies to Enhance the Anti-tumor Effects of MSCs

Formation of new blood vessels (angiogenesis) has been regarded as one of the key factors in the progression of cancers. Treatment with anti-angiogenetic agents appears to become one promising approach in cancer therapy. MSCs have been considered as effective vehicles to deliver such factors. Several lines of evidence have shown the use of genetic modification to generate MSCs that are capable of sending anti-angiogenetic factors to the tumor sites. For example, MSCs overexpressing endostatin have been reported to be effective in controlling the growth of ovarian cancer. It was shown that the beneficial effect was likely due to the inhibition of neovascularization, enhanced apoptosis, and inhibition of cell proliferation (Zheng et al., 2012). Equally important, the fact that MSCs can be engineered to express intact or single-chain antibody fragments (Frank et al., 2010) has opened the possibility to generate MSCs that are capable of secreting anti-angiogenetic antibodies, such as an anti-VEGF antibody.

Another strategy in the development of anti-cancer MSCs is by using these cells to deliver oncolytic adenovirus. One example of this strategy is the use of MSCs to carry and deliver the Delta-24-RGD adenovirus, which has a potent anti-glioma property (Yong et al., 2009). Experimental evidence in mice has demonstrated the efficacy of this approach in eradicating brain glioma (Yong et al., 2009).

#### Genetic Engineering and the Pro-tumorigenic Effect of MSCs

One important aspect of MSC-based therapy that may hamper the clinical translation is the capability of MSCs to promote cancer progression. Co-injection of MSCs with cancer cells *in vivo* can induce the growth of the tumor cells. This has been observed in models of ovarian (Spaeth et al., 2009), prostate (Jung et al., 2013), colorectal (De Boeck et al., 2013), and breast cancer (Karnoub et al., 2007). It is thought that the pro-tumorigenic effects of MSCs might be due to the cell-to-cell contact and/or secretion of paracrine factors (Babajani et al., 2020). *In vitro* studies showed that co-culturing of primary cancer cells with bone marrow-derived MSCs significantly reduced the apoptosis of the cancer cells. Direct cell-to-cell contact was proposed as the anti-apoptotic mechanism, although the ligands and/or the receptors that are responsible for the interaction are not known yet (Lee et al., 2019).

Equally important, several lines of evidence have suggested that MSCs that are recruited to the tumor microenvironment might secrete paracrine factors and cytokines that can facilitate tumor growth, angiogenesis, and survival against anti-cancer drugs. For example, injected MSCs to cancer sites produce pro-growth factors such as insulin-like growth factor, platelet-derived growth factor, and epidermal growth factor receptor (Akimoto et al., 2013), as well as cytokines and factors like IL-6 (Lin et al., 2013) and TGF-b (Ye et al., 2012). Interestingly, the secretory profiles of MSCs recruited to the tumor sites are different from those of primary MSCs outside the tumor microenvironment, suggesting that the pro-tumor phenotype might be due to the local niche produced by the tumor cells (Babajani et al., 2020).

It is important to note that in the majority of observations showing the pro-tumorigenic effects of MSCs, the researchers used native MSCs in their models. By contrast, most of the studies using genetically manipulated MSCs as outlined above have reported significant anti-tumor effects as opposed to the pro-tumorigenic effect. This suggests that genetic modification might be a promising strategy to control the pro-tumorigenic effects of MSCs. In addition, drug-loading has been demonstrated to be an effective approach to control the pro-tumorigenic phenotype. For example, drug priming with paclitaxel has been shown to improve the anti-tumor activity of MSCs and induce the anti-angiogenic effect by inhibition of ICAM1, VCAM1, and VEGF expressions (Pessina et al., 2011).

### The Use of Engineered MSC to Treat Non-cancer Conditions

**FIGURE 3:**
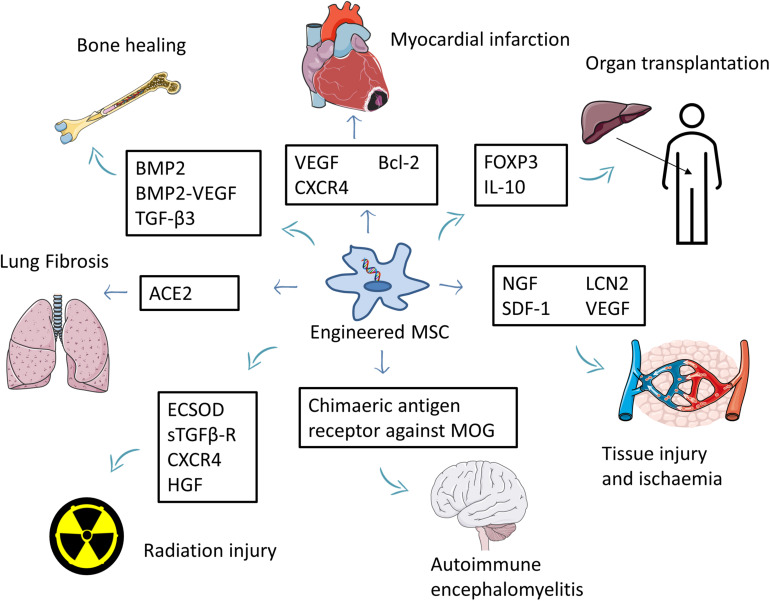
Summary of the use of engineered MSCs for the treatment of non-cancer diseases. MSCs overexpressing beneficial factors are used to treat several conditions such as myocardial infarction, tissue injury, ischemia, and autoimmune encephalomyelitis. Engineered MSCs were also used to improve bone healing and reduce the side effects of organ transplantation. Part of this figure was created using images from Servier Medical Art Commons Attribution 3.0 Unported License. (http://smart.servier.com). Servier Medical Art by Servier is licensed under a Creative Commons Attribution 3.0 Unported License.

Engineered MSCs were used to treat various non-cancer conditions, including allogeneic liver transplantation (Niu et al., 2014; Qi et al., 2015), autoimmune encephalomyelitis (Fransson et al., 2014), nerve injury (Wang et al., 2015b), wound healing (Nakamura et al., 2013), critical limb ischemia (Beegle et al., 2016), acute kidney injury (Roudkenar et al., 2018), myocardial infarction (Li et al., 2007; Huang et al., 2010; Zhang et al., 2010; Zhu et al., 2012), various bone defects (Chang et al., 2003, 2004; Chang et al., 2010; Tsuchida et al., 2003; Jiang et al., 2009; Lin et al., 2010, 2011, 2012a,b; Lin et al., 2015; Zhao et al., 2010; Fu et al., 2015), lung fibrosis (Min et al., 2015), radiation-induced toxicity (Abdel-Mageed et al., 2009; Xue et al., 2013; Zhang et al., 2014; Wang et al., 2015a; Zhang et al., 2019), Huntington’s disease (Dey et al., 2010), and rheumatoid arthritis (Liu et al., 2013; Dong et al., 2020; Figure 3 and Supplementary Table 2).

#### Engineered MSCs to Prevent Rejection in Organ Transplantation

A study using a mouse model of allogeneic liver transplantation used engineered MSC expressing transcription factor fork head box P3 (Foxp3) to increase the median survival time of the animals receiving organ transplantation. Foxp3 caused an increase in Treg level, which has a strong immunomodulatory effect. The increase was T cell contact dependent and was associated with the upregulation of programmed death ligand 1 (PD-L1) expression in MSC (Qi et al., 2015). Another study used interleukin-10 secreting MSCs (MSC-IL10) to prevent rejection in an allogeneic liver transplantation model. It showed an increase in median survival time of transplanted animals compared to those injected with non-engineered MSCs, though non-engineered MSCs also demonstrated increased median survival time compared to control (non-MSC-injected animals) (Niu et al., 2014). This might be due to the native immunomodulation effect of MSC (Andreeva et al., 2017). Secretion of IL10 might increase the levels of Treg-associated anti-inflammatory cytokines (IL-10 and TGF-β1) and decrease Th17-associated pro-inflammatory cytokines (IL-17, IL-6, IFN-γ, TNF-α, and IL-23) (Niu et al., 2014).

#### Engineered MSCs for the Treatment of Autoimmune Encephalomyelitis

Engineered MSCs have been used in a study using a mouse model of experimental autoimmune encephalomyelitis (EAE). Fransson et al. (2014) examined the use of genetically engineered MSCs expressing a myelin oligodendrocyte glycoprotein (MOG) specific receptor (MSC-CARα-MOG). Anti-rat MOG antibodies cloned from a single-chain variable fragment (scFv) were introduced into a chimeric antigen receptor (CAR), producing a CARαMOG construct. This was then inserted into a lentiviral vector. They discovered that the engineered MSCs exerted beneficial effects in controlling the disease and showed that the intranasal MSC application route was better than intraperitoneal injection. Intranasal route of MSCs treatment caused symptom free at days 27–30. Non-engineered MSCs also caused reduced experimental autoimmune encephalomyelitis as indicated by a better EAE score compared to control when given via an intranasal route, but the improvement was not as good as those treated with MSC-CARα-MOG. When given via an intraperitoneal route, non-engineered MSCs showed a similar EAE score to that of control. The beneficial effect of MSC-CARαMOG might be due to the modulation of the inflammatory cytokines released by T-lymphocytes in the brain. In EAE, activated T-cells migrate across the blood–brain barrier to the brain parenchyma, resulting in brain inflammation, myelin loss, and axon damage. T-cells recovered from the brain of EAE mice produced a lower level of interferon-ɤand higher level of interleukin-17 when treated with MSC-CARαMOG compared to those treated with control MSC. Interestingly, T-cells isolated from the spleen did not show this phenotype, indicating a tissue-specific effect (Fransson et al., 2014).

#### Engineered MSCs for Treating Tissue Injuries and Ischemia

Wang and co-workers generated engineered MSCs that secrete nerve growth factor (NGF) and used them to treat nerve injury in a rabbit model. They showed that treatment with these cells (MSC-NGF) increased the formation of nerve fibers and the density of myelinated nerve fibers. It also decreased the deposition of myelin debris compared to control animals. The study demonstrated that the MSC-NGF cells enhanced the nerve regenerative capacity (Wang et al., 2015b).

Another study used stromal cell-derived factor-1 (SDF-1) secreting MSCs (MSC-SDF-1) to treat full thickness wound in a rat model. Wound closure and blood vessel formation were significantly improved in the MSC-SDF-1-treated group. Animals treated with non-engineered MSCs have also shown improvement compared to the control group, although not as significant as in the MSC-SDF-1-treated group. SDF-1 increased vascular endothelial growth factor (VEGF) expression, which induced angiogenesis. Furthermore, there was an increase in dermal fibroblast migration in treated animals (Nakamura et al., 2013).

The study by Beegle et al. (2016) used engineered MSCs expressing VEGF in a mouse model of critical limb ischemia. The results showed that mice treated with these MSCs displayed better blood flow at week 10 after treatment compared to the control group. This finding is expected as VEGF is known as a strong angiogenesis factor. It is important to note that allogeneic MSCs for critical limb ischemia have been approved for clinical use in India (Robb et al., 2019).

Using a mouse model with acute kidney injury, a study used lipocalin-2-secreting MSCs (MSC-Lcn2). The results showed that the number of cast and tubular necrosis was significantly decreased compared to mice treated with non-engineered MSCs and with the control groups. At day 21 following treatment, the serum creatinine level in the MSC-Lcn2-treated group decreased significantly to a level comparable to those of normal mice. The blood urea nitrogen level was also decreased, although the level was still higher than normal. Lipocalin-2 has been shown to upregulate the expression of various growth factors, such as hepatocyte growth factor (HGF), insulin-like growth factor, fibroblast growth factor, and VEGF. All of them have the capability to trigger kidney regeneration. This was indicated by the increase in the expression of kidney regenerative markers (i.e., the proximal tubular epithelium markers such as AQP-1 and CK18) and the decreased expression of kidney injury markers such as KIM-1 and cystatin C (Roudkenar et al., 2018).

#### Engineered MSCs for the Treatment of Myocardial Infarction

Our systematic literature search has indicated four studies on the use of engineered MSCs to treat myocardial infarction. One study was conducted using a mouse model, and the others used a rat model. The first study used VEGF-secreting MSCs and showed that treatment with these cells improved heart function compared to the control group. It was reported that the improvement was likely due to the increased in capillary density because VEGF is known as a potent angiogenic factor (Zhu et al., 2012).

Two studies used engineered MSCs that produce and secrete CXCR4 (MSC-CXCR4) (Huang et al., 2010; Zhang et al., 2010). In a study reported by Zhang et al. (2010) MSC-CXCR4 cells were implanted as cell sheets, and Huang et al. (2010) grew MSC-CXCR4 on the animals’ peritoneum and then applied them onto the scarred myocardium. Both approaches resulted in the improvement of heart function compared to the control group. CXCR4 increased cell engraftment and angiogenesis as well as reduced myocardial remodeling, hence improving heart function (Huang et al., 2010; Zhang et al., 2010). MSC-CXCR4 was also used in combination with diprotin A, a molecule that can inhibit the enzymatic degradation of the SDF-1α-CXCR4 complex by the enzyme CD26/dipeptidyl peptidase IV (DPP-IV). This would result in the prolongation of the CXCR4 activity. Treatment with the combination of MSC-CXCR4 and diprotin A produced better therapeutic effects compared to treatment with MSC-CXCR4 alone (Zhang et al., 2010).

The fourth study in the field of myocardial infarction indicated by our literature search involved the use of engineered MSCs overexpressing Bcl2 (MSC-Bcl2). Bcl2 is known as a potent anti-apoptotic agent. In this study, Li and co-authors demonstrated that treatment with MSC-Bcl2 resulted in the improvement of heart function following myocardial infarction compared to the control group. Bcl2 was shown to induce cell engraftment and VEGF expression level, which, in turn, increased angiogenesis and eventually improved heart function (Li et al., 2007).

#### The Use of Engineered MSCs to Induce Bone Healing

We found 13 studies on the use of genetically engineered MSCs for the treatment of various types of bone defects (Chang et al., 2003, 2004; Chang et al., 2010; Tsuchida et al., 2003; Jiang et al., 2009; Lin et al., 2010, 2011, 2012a,b, 2015; Lin et al., 2013; Zhao et al., 2010; Fu et al., 2015). In most studies, bone morphogenetic protein 2 (BMP-2) was overexpressed in the MSCs, either alone (Chang et al., 2003, 2004; Chang et al., 2010; Tsuchida et al., 2003; Jiang et al., 2009; Zhao et al., 2010) or in combination with VEGF (Lin et al., 2010, 2011, 2012a,b, 2015; Fu et al., 2015). Whilst in one study BMP2 overexpression was compared with TGF-β3 overexpression (Lin et al., 2013). All studies reported improvement in bone healing following implantation of engineered MSCs. This is not unsurprising as BMP2 is known as a strong inducer of osteogenesis (Chang et al., 2003, 2004; Chang et al., 2010; Tsuchida et al., 2003; Jiang et al., 2009; Lin et al., 2010, 2011, 2012a,b, 2015; Lin et al., 2013; Zhao et al., 2010; Fu et al., 2015). On the other hand, VEGF helped the process by promoting vascular network formation and hence enhanced the BMP2 osteogenesis effect (Lin et al., 2010, 2011, 2012a,b, 2015; Fu et al., 2015).

A study examined a combination treatment of MSC-BMP2 with the addition of various tissue scaffolds, such as ultrapure alginate, alginate, arginine–glycine–aspartic acid (RGD)–alginate, and collagen-1 (Chang et al., 2010). The authors reported that the best scaffold was the type 1 collagen as it degraded faster and improved bone regeneration. By contrast, RGD alginate was considered inferior because it inhibited initial chondrogenesis (Chang et al., 2010).

Tsuchida et al. (2003) compared the use of syngeneic MSCs with allogeneic MSCs in a rat model of bone defect. They showed that syngeneic MSCs exhibited better therapeutic effects than allogeneic MSCs. However, when the allogeneic MSCs were transfected with BMP2 and then implanted in combination with an immunosuppressant drug FK506, they found improvement of the therapeutic effects of the allogeneic MSCs to the level similar to that of syngeneic MSCs. This effect could be attributable to the BMP2 expression of the engineered allogeneic MSCs.

Several other studies examined the differential effects of the transient and constitutive expression of the BMP2 and VEGF combination in the MSCs. The data indicated that the constitutive expression of the osteogenic factor showed better therapeutic effects (Lin et al., 2011, 2012a).

BMP2-engineered MSCs seemed to produce better therapeutic effects compared to TGF-β3-engineered MSCs in a rabbit model of calvarial bone defect. A study reported that implantation of BMP2-engineered MSCs promoted bone formation and significantly improved bone volume and bone density. The better therapeutic effect of these cells was likely due to the ability of BMP2 to promote osteogenesis and chondrogenesis as well as to stimulate the formation of the dura mater, which is important in calvarial bone healing. The study also compared the use of two types of tissue scaffolds, i.e., poly(L-lactide-co-glycolide) (PLGA) and gelatin. It was shown that the gelatin sponge produced better chondroinductive ability than PLGA and subsequently improved bone healing (Lin et al., 2013).

A recent review from Freitas et al. summarized the diverse approaches in using genetically modified MSCs for the treatment of critical-size, non-union, and delayed bone defects. Typically, the MSCs were engineered with genes responsible for osteogenesis (e.g., TGF-β, BMPs, core binding factor α1/Cbfa1, and Osteorix/Osx), angiogenesis (e.g., VEGF), anti-apoptosis (e.g., human telomerase/hTERT), or non-coding RNA (microRNAs). The MSCs can be implanted in the lesions, or alternatively, the secretomes can be given either locally or systemically. The critical questions in utilizing engineered MSCs are how to ensure the safety of the genetic modification and how to standardize the end products of the therapy (Freitas et al., 2019).

#### The Use of Engineered MSCs to Improve Lung Fibrosis

A study showed that MSCs overexpressing angiotensin-converting enzyme 2 (ACE 2) produced a better therapeutic effect in a bleomycin-induced lung fibrosis model in mice (Min et al., 2015). Mice treated with the MSCs-ACE2 cells showed a significantly better lung phenotype compared to mice treated with ACE2 only or with non-engineered MSCs (Min et al., 2015; Supplementary Table 2). This finding indicated the synergistic actions of MSCs and ACE2. MSCs are known to have fibrinolytic properties (Srour and Thebaud, 2015), whereas ACE2 has an anti-fibrotic activity via the degradation of angiotensin 2 (Ang 2), which is known as a pro-fibrotic agent (Min et al., 2015).

#### Engineered MSCs to Alleviate Radiation-Induced Toxicity

Another condition that has been successfully treated by genetically engineered MSC is radiation-induced toxicity. A study using a mouse model receiving total body irradiation showed that genetically modified MSCs overexpressing extracellular superoxide dismutase (ECSOD) could alleviate the toxic effects of the radiation, resulting in an increase in the 35-day survival rate to 52%, which was significantly better compared to control (10%). The mechanism of action was likely due to the improved scavenging process of the superoxide anion by ECSOD, which might prevent hematologic toxicity (Abdel-Mageed et al., 2009).

Similarly, a study using a radiation-induced lung injury model in mice demonstrated that treatment with MSCs expressing soluble transforming growth factor-ß type II receptor (sTGF-ß-R) resulted in significantly higher survival (80%) compared to mice treated with native MSCs (40% survival rate), and none of the control animals (no MSC treatment) survived. It is believed that the fibrinolytic effect of MSC in combination with the anti-inflammatory effect of sTGF-ß-R is responsible for preventing inflammatory cell infiltration, pro-inflammatory cytokine secretion, and collagen overproduction in mice treated with MSC-sTGF-ß-R (Xue et al., 2013).

In keeping with those results, another study using a mouse model of radiation-induced lung injury analyzed the use of MSCs overexpressing CXCR4. The genetically engineered MSCs displayed better performance compared to native MSCs as CXCR4 expression facilitated MSC migration and homing to the injury sites. CXCR4 is a receptor for stem cell stromal cell-derived factor-1 (SDF-1), and the activation of CXCR4 by SDF-1 modulates the signaling pathway important for stem cell migration and homing (Zhang et al., 2014).

Two other studies related to radiation-induced toxicity involved the use of HGF. Wang et al. showed that MSCs expressing HGF showed a significantly better therapeutic capacity in alleviating radiation-induced intestinal injury compared to native MSCs. It is thought that the regenerative and anti-apoptotic effects of HGF might be the responsible factors in enhancing intestinal epithelial cell regeneration (Wang et al., 2015a). MSCs overexpressing HGF were also used in radiation-induced liver damage. These cells have been shown to produce better regenerative effects in the liver (Zhang et al., 2019).

#### The Use of Genetically Engineered MSC for the Treatment of Huntington’s Disease and Rheumatoid Arthritis

Huntington’s disease is caused by mutation in the huntingtin (HTT) gene resulting in the production of mutant huntingtin (mutHTT) protein. mutHTT may induce the death of the medium spiny neurons in the brain striatum. The mechanism of neuronal cell loss involves the reduction of neurotrophic factors, such as brain-derived neurotrophic factor (BDNF). A study using a mouse model of Huntington’s disease demonstrated that treatment with MSCs overexpressing BDNF and/or NGF produced better therapeutic effects compared with treatment with naive MSCs (Dey et al., 2010).

A study on rat and mouse models of arthritis showed that genetically modified MSCs expressing human soluble tumor necrosis factor receptor type 2 (hsTNFR) could partially prevent arthritis symptoms and produced better therapeutic effects than naive MSCs. However, MSC-based treatment was still less effective than treatment with dexamethasone. In addition to the intrinsic anti-inflammatory properties of the MSCs, the expression of hsTNFR might inhibit the pro-inflammatory effects of TNF-α. hsTNFR could bind and decoy TNFα and thus block the TNF-α-mediated inflammatory effect (Liu et al., 2013).

More recently, MSCs expressing HGF were reported for the treatment of rheumatoid arthritis in mice. The study showed that MSC-HGF could alleviate arthritis symptoms, but at the later phase of the disease, the effect was not better than treatment with naive MSCs. HGF at the early phase of the disease displayed an immunosuppression effect, but at a later phase, HGF induced the activation of fibroblast-like synoviocytes. These cells had the capacity to produce Il-6, which induced inflammation, cell proliferation, and decreased apoptosis (Dong et al., 2020).

## Conclusion

Engineered MSCs have been used successfully to treat various diseases and conditions in animal models. However, it is important to note that animal models might not be similar to human diseases/conditions. Therefore, the results should be interpreted cautiously as a translational study in human so far did not show any positive result. Nevertheless, MSC-based therapy is still a promising new therapeutic modality for various conditions, and it is clear that enhancement of its therapeutic effects can be achieved by inducting the expression of beneficial paracrine factors. Further studies are still needed to accelerate the pre-clinical findings into clinical application.

## Author Contributions

JP conceived the idea, performed the literature search and data entry on tumor/malignancy treatment, analyzed and compiled the data, and wrote the manuscript. TB performed a review on cancer treatment. WM performed a review on engineered MSCs to prevent rejection in organ transplantation, treatment of autoimmune encephalomyelitis tissue injuries, ischemia, and myocardial infarction. RA performed a review on a metastatic cancer model. RN performed the data entry and a review on engineered MSCs for bone regeneration. ID conceived the main idea and performed a review on clinical trials. DO conceived the idea, compiled the data, and wrote and edited the manuscript. All authors contributed to the article and approved the submitted version.

## Conflict of Interest

The authors declare that the research was conducted in the absence of any commercial or financial relationships that could be construed as a potential conflict of interest.
