# Supplementary material for: Enhancement of the Therapeutic Capacity of Mesenchymal Stem Cells by Genetic Modification: A Systematic Review
Source: Front Cell Dev Biol. 2020 Oct 30;8:587776. doi: 10.3389/fcell.2020.587776 (PMC7661472; doi:10.3389/fcell.2020.587776)
Supplement: Supplementary file 1 [file Table_1.docx]

**Table S1. List of studies using genetically engineered MSCs for cancer treatment**

| **Reference** | **Disease/disease models** | **Species/number of subject** | **MSC source and passage, treatments** | **Vector/**  **gene insertion /expressed proteins/drug** | **Outcome** | **Adverse event** | **Mechanism of action** |
| --- | --- | --- | --- | --- | --- | --- | --- |
| (Segaliny et al., 2019) | Breast Cancer- bone metastasis | BALB/c J  mice  C= 22  T= 18 | Hu BM-MSC, Pa: NR.  C= PBS/MSC+5FC/ 5FU  T= MSC-gene +5FC 1x, 3x | mRNA-lipofectamine/ PSGL-1/ SLEX,  CD, OPG | Best Survival: T 3x | Minimal toxicity >< chemo (5FU) | Homing factor 🡪tumor blood vessels  Conversion: 5-FC🡪 5-FU  Osteolysis inhibition |
| (Fritz et al., 2008) | Bone (tibia) metastasis of prostate cancer | Male SCID/Bg mice C= 7  T1= T2= T3= 15 | C3-MSC, Pa= NR  Co-injection PC3 with:  C= C3-MSC= 1:3  T= C3-MSC-ATF= 1:3/1:1/3:1 | Adeno virus/ hATF | tumor growth-  d-14, d-21,  tumor vascular network d-21,  New bone formation-d-14: T(1:3) the best | NR | hATF:  anti- invasion, anti-migration, anti-angiogenesis  MSC: new bone formation |
| (Sasportas et al., 2009) | Glioma (GBM8) mouse model: | SCID Mice  C= 9  T= 9 | Hu BM-MSC, Pa: NR  Implantation/co-injection GBMB IC with:  C= MSC  T= MSC-s-TRAIL | Lentiviral vector/ s-TRAIL | Glioma cell T<C  Survival T>C  Act-Cas3:  T1> C | NR | s-TRAIL🡪 apoptosis |
| (Altanerova et al., 2012) | Glioblastoma (C6) rat model | Male SD rat  C=8 T= 21 | Hu AT-MSC, Pa: NR  Co-injection C6 IC-with:  C= 5FC-IP/MSC+5FC-IVe  T= MSC-CD (1:2) CL+ 5FC-IP/ MSC-CD (1:0.2) CL + 5FC-IP/MSC-CD (1:4) + 5FC-IVe/MSC-CD (1:4)+5FC–Ive (2xT) | Retrovirus/ CD-UPRT | Survival:  Best: MSC-CD (1:4) + 5FC -Ive (2xT) | (-) | Conversion: 5-FC🡪 5-FU |
| (Altaner et al., 2014) | Glioblastoma (C6) rat model | Adult male CD® IGS rats C1= 37  T= 64 | hu BM-MSC/hu AT-MSC, Pa: NR  C= (-)/ d-13-R/ d-13R + BM IC+ 5FC –IVeP/ d-10 R  T= d-5 - AT-CD-IC+ 5FC IVeP/ d-5, d-14 AT-CD-IC +5FC-IVeP/ d-5- AT-CD-P + 5FC –IveP/ d-5 - d-14-AT-CD-P + 5FC –IVeP/ **d-13-R -d13, d-21+ BM-CD -IC+ 5FC –IveP**/ d-10 R, d-10, d-17 AT-CD-IC - 5FC –IveP/ d11-R, d-11, d-24 + AT-CD-IC+ 5FC -IVeP | Retrovirus/ CD-UPRT | Best 150 day survival: d-13-R -d13, d-21+ BM-CD -IC+ 5FC –IveP | (-) | Conversion of pro drug 5-FC🡪 5-FU |
| (Fei et al., 2012) | Glioma (C6)  rat model | SD rats C= 8  T= 14 | Rat BM-MSC, Pa= P3  Co-injection d-0-C6-IC with:  C= 5FC-IC (d7-14)  T= MSC-CD (1:1)+ 5FC-IC (d7-14) / MSC-CD (1:2)+ 5FC-IC (d7-14) | Lentiviral vector/ CD | Smallest tumor size, longest survival, highest apoptosis: MSC-CD (1:2) + 5FC | NR | Conversion: 5-FC🡪 5-FU |
| (Kosaka et al., 2012) | Glioma (9L)  rat model | (balb/c- nu/nu mice (SC)  C= 8  T= 4  Fischer-344 rats (IC)  C= NR  T= NR | Rat BM-MSC, Pa= NR  **Co-injection 9L SC with:**  C= MSC+PBS/MSC+ 5FC-IPd2-14  T= MSC-CD+5FC IP d2-14  **Tumor injection:**  C= PBS/MSC IT+PBS/ MSC IT + 5FC IP d2-14/ MSC-CD IT+PBS  T= MSC-CD IT+ 5-FC-IP-d2-14 | Adenovirus vector/ CD | Co-injection: smallest tumor size d-21, d-28, d-36:  MSC-CD + 5-FC  Tumor: longest survival: MSC-CD + 5-FC | 5FC systemic toxicity (-) | Conversion: 5-FC🡪 5-FU |
| (Chung et al., 2016) | Glioma  (U87MG)  Mouse model | BALB/c nude mice  C= 10  T= 10 | Hu BM-MSC, Pa= NR  C= 5-FC-IP 5x/wk  T= MSC-CD-IC+ 5-FC –IP- 5x/wk | Retroviral vector/ CD | Tumor size:  T < C (T↓, C↑)  Survival: T > C  Ki67: T < C  Apoptosis: T > C | NR | Conversion: 5-FC🡪 5-FU |
| (Chang et al., 2010a) | Glioblastoma (C6) rat model | Adult male SD albino rats  C= 32  T= 24 | Hu BM-MSC, Pa= NR  Co-injection C6 IC with:  C= PBS IC/PBS IC+d-2-5FC50-IP/ PBS IC+d-2-5FC500–IP/ MSC-CD (H)-IC  **T**= MSC-CD-IC+d-2-5FC50-IP/ **MSC-CD IC+d-2-5FC500 IP**/ MSC-CD- IC+d-25FC500–IP repeat d-7, d-14/ d-3 MSC-CD-IC +d-4 5FC500 IP, repeat d-10, d-17 | Retrovirus/ CD | Smallest tumor size: MSC-CD IC+d-2-5FC 500 IP | NR | Conversion: 5-FC🡪 5-FU |
| (Bak et al., 2011) | Glioma (U87MG) mouse model | Balb/c athymic, nude mice  ATS:  C= 16  T= 8  ACLS:  C= 20  T= 10 | Hu ESC derived MSC  Pa= P13-P20  **Bacvec:** ATS  C= PBS+ GCV/ MSC+GCV  T= MSC-TK+ GCV  **d-5-Lenvec**: ACLS  C= PBS+GCV/ MSC+ GCV  T= MSC-TK+ GCV | Rec Baculo virus or Rec Lentivirus/ HSV-TK | ATS: Tumor size  d11, d-25: T< C  ACLS: Tumor necrosis: T> C  ATS, ACLS: Survival:  T>>C | NR | HSV-TK: GCV phosphorylated🡪 toxic  MSC: Homing  Lentiviral broadened expression window vs baculovirus vector |
| (Martinez-Quintanilla et al., 2013) | Glioblastoma multiforme  (Gli36vIII) mouse model | Scid/nude mice C= 14  T= 12 | Mouse MSC  Source, Pa: NR  C= PBS IC/ PBS IC+ GCV-IP – 10d  T= MSC-TRAIL-IC +GCV IP 10d/ MSC-TRAIL-TK -IC +GCV IP- 10d | Lentiviral and retroviral vector/ HSV-TK,  s-TRAIL | Tumor growth: T< C  Median survival:  MSC-TRAIL +GCV (39d)> MSC-TRAIL -TK +GCV (31.5d)> C (17 d) | NR | HSV-TK 🡪 GCV– phosphorylated🡪 toxic  s-TRAIL 🡪apoptosis |
| (Suryaprakash et al., 2019) | Solid tumor  Glioblastoma multiforme (U87MG) mouse model | Nu/J nude mouse C= 6  T= 3 | Hu MSC spheroid  MSC source: NR  Pa= P3-P6  C= PBS/MSC+MTX NC-ET  T= MSC hybrid spheroids –TRAIL - MTX –NC-ET | PEGylated DNA-templated NC system / TRAIL, PL against IL13Rα2, MTX | Tumor size:  d-21: T< C  T= 14% C (PBS) (0.053 g vs 0.360 g ) | Histology: No significant organ toxicity | TRAIL🡪apoptosis  MSC-PL against IL13Rα2 🡪 homing  MTX🡪anticancer drug |
| (NguyenThai et al., 2015) | Solid tumor  Osteosarcoma (Cal 72) mouse model | Nude mice  C= 5  T= 5 | Hu BM-MSC  Pa: P2-P3  Co-injection Cal72 SC with:  C= 5FC IP d2-4-6  T= MSC-CD+5FC IP-d2-4-6 | pEGFP- vector/  Yeast CD-UPRT | Tumor size:  C= 118.43 ± 23 mm^3^  T= 69.7 ± 21 mm^3^ (p< 0.05).  Metastasis (lung):  C=4, T= (-) | (-) | Conversion: 5-FC🡪 5-FU |
| (Kucerova et al., 2007) | Solid tumor  Colon adenocarcinoma (HT-29) mouse model | Athymic nude mice  (BALB/c-nu/nu)  Co-inj  C= 21  T= 12  Tumor T  C= 12  T= 6 | AT-MSC, Pa= P5  **Co injection HT-29 SC with:**  C= 5FC-IP-10x/ MSC 1:1+5FC-Ip-10x/ MSC 10:1+5FC-IP-10x  T= MSC-CD 1:1+5FC-IP 10x/ MSC-CD 1:0.1+5FC-IP 10x  **Tumor** **treatment**  C= PBS IV+ 5FC-IP-10x/ MSC IV+5FC-IP-10x  T= MSC-CD1:4 IV + 5FC-IP-10x | pST2 retrovirus + protamine sulfate/ CD-UPRT | **Co-injection:**  Tumor size d-5, d-7, d-10, d-12: T-HD= T-LD <C  tumor weight d-18: T-HD < T-LD < C  **Tumor injection -**  Tumor size d-10, d-14, d-18: T < C | (-) | Conversion: 5-FC🡪 5-FU |
| (Schug et al., 2018) | Solid tumor  Hepatocellular carcinoma (HuH7) mouse model | CD1 nu/nu mice (Charles River) C= 4  T= 8 | Primary Hu BM-MSC  Pa= NR  Tumor Irradiation:  C= 0 Gy  T= 2 Gy/5 Gy  d-2: MSC-NIS tail vein  d-4: 0.5 mCi 123I IP | CMV-NIS-pcDNA3/ NIS | MSC-NIS engraftment 5Gy> 2Gy > C  Radioiodide accumulation in tumor: 5Gy> 2Gy> C | Radioiodide accumulation in salivary gland, thyroid, stomach and bladder | NIS🡺uptake radioiodide by MSC  External beam radiation 🡪homing of MSC to tumor |
| (Nouri et al., 2015) | Solid tumor  Ovarian cancer (SKOV3) mouse model | nude mice  C= 35  T= 15 | Hu BM-MSC, Pa= NR.  C= PBS-IT/ MSC-TK- IT/ MSC-CD- IT/ MSC-NTR-IT/  GCV-IT/ 5-FC –IT/ CB1954-IT  T= MSC-TK- IT + GCV/ **MSC-CD- IT+ 5-FC/** MSC-NTR- IT+ CB1954 | pBudCE4.1 (dual promoter mammalian expression vector)/ HSV-TK-SR39  Yeast CD-UPRT  NTR | Tumor size:  d-18: smallest in **MSC-CD- IT+ 5-FC=** 5-FC –IT  d-36:smallest in  **MSC-CD- IT+ 5-FC**< MSC-NTR- IT+ CB1954= CB1954-IT | NR | HSV-TK-SR39: GCV phosphorylated🡪 toxic  CD-UPRT: 5-FC🡪 5-FU  NTR: CB 1954 🡪 cytotoxic form |
| (Amara et al., 2016) | Solid tumor  Lung epithelial tumor (TC1) mouse model | C57BL/6  mice Co-Inj:  C= 22  T= 24  Tumor inj  C= 21  T= 15-3 | Murine BM MSC from C57BL/6 mice, Pa= NR  **Co-injection TC1 with:**  C= PBS-IP/ CPA-45 IP/ CPA- 90 IP/ CPA-140 IP  T= TC1-Cyp +PBS-IP/ TC1-Cyp + CPA- 45 IP/ TC1-Cyp + CPA- 90 IP/ TC1-Cyp + CPA- 140 IP  **Tumor injection:**  C= (-)/ CPA-90 IP/ MSC-IT/ MSC-IT + CPA-90 IP  T= MSC-Cyp-IT/ MSC-Cyp-IT + CPA –90 IP | pHIV-EF1L-thy1/GFP-W lentiviral vector/ CYP2B6TM | Co=injection:  Tumor size d-20, d-40, d-70: TCI-Cyp + CPA- 90 IP = TCI-Cyp + CPA- 140 IP < C  Tumor injection:  Tumor size d-42, d80: MSC-Cyp-IT + CPA –90 IP (n=3) << (n=6) = MSC-IT + CPA-90 IP | 3/12 MSC-Cyp dead (anaphylactic shock ??) due to residual FCS in MSC-Cyp | Cyp: CPA 🡪 toxic form |
| (Krassikova et al., 2016) | Solid tumor  (LLC) lung carcinoma mouse model | C57BL/6 mice  C= 8 -10  T= 32-40 | Syngeneic ATMSC  Pa= NR  C= PBS-IT/ d-7-AT-MSC-IT/ d-14 Lysomustine IP  T= d-7- AT-MSCs-CD –UPRT-IT+ 5FC –IP (d8-14)/ d-7- AT-MSCs-CD-UPRT-VP22-IT+5FC-IP (d-8-14)/ d-7-AT-MSCs-CD-UPRT -IT+ 5FC –IP (d-8-14)+ d-14-Lysomustine IP | pCpGfree-mcs + electroporation  / CD-UPRT, VP22 | Tumor growth inhibition: d-7-AT-MSCs-CD- UPRT + 5FC (d-8-14) + d-14 Lysomustine IP (86%) > d-7- AT-MSCs-CD-UPRT + 5FC (d-8-14)= d-7- AT-MSCs-CD-UPRT-VP22 + 5FC (d-8-4)= 56% > C  Increase life span:  Best AT-MSCs- CD- UPRT + 5FC (d-8-14) + d-14 Lyso-mustine IP= 60% | NR | Conversion: 5-FC🡪 5-FU  5FU-lysomustine synergy  VP22: facilitate secretion and re-uptake of  fusion proteins to neighboring cells, |
| (Toro et al., 2016) | Solid tumour  and metastatic ovarian cancer  (SKOV-3, A2780) mouse model | Athymic nude mice (Balb/c  nu/nu)  C= 19-28  T= 19-28 | Hu AT-MSC, Pa= NR  **Co-inj SKOV-3 (SC) with:**  C=(-)  T= MSC-CD 1:0.1 + 5-FC –IP (d-3-14)  **Co-inj A2780 cells (SC) with:**  C= (-)/ MSC 1:0.2  T= MSC-CD 1:0.2 + 5-FC –IP (d-0-14)  **Metastasis SKOV-3 -IP:**  C= (-)  T= MSC-CD 1:0.4 IP (d-7,d-14,d-21)+ 5-FC IP (d-7-21)  **Metastasis A2780 - IP:**  C= (-)  T= MSC-CD 1:0.4 IP (d-7,d-14,d-21) +5-FC -IP(d-7-21) | pST2 retrovirus/ Yeast CD-UPRT | Tumor size:  Co-inj SKOV-3 -d-0: T< C  Co-inj A2780 cells: T< C  Metastasis SKOV-3 cells: T= C  Metastasis A2780 cells: T< C  120d disease free survival:  Metastasis A2780 :  T= 33%  . | NR | CD::UPRT: 5-FC🡪 5-FU |
| (Zou et al., 2012) | Solid tumor  murine breast tumor  (TUBO  cell line) mouse model | BALB/c mice  Prevention  C= 16  T= 8  Tumor inj  C= 16  T= 8  CD4/CD8 T deletion =3  Blocking LIGHT =3 | BALB/c BM-MSC, Pa= NR  **Prevention:**  C=d-0 PBS/ d-0 MSC SC  +d-13 TUBO 2.5:1 ACLS SC  T= d-0 MSC-L SC + d-13 TUBO 2.5:1 ACLS SC  **Tumor inj:**  C= d-0 TUBO SC + d-7 PBS/ d-7 MSC 1:2.5 ACLS SC  T= d-0 TUBO SC+ d-7 MSC-L 1:2.5 ACLS SC  **CD4/CD8 T cell deletion:**  anti-CD8 SC  anti-CD4 SC  **Blocking LIGHT**:  **LTβR-Ig** 100 mg IP | Retrovirus/  LIGHT | **Prevention:**  Tumor size-d-15-30: T= (-) << d-20-C=100-200mm^3^, d-30-C=1400 mm^3^  d-50 survival: T=100% >> C= 0%  **Tumor inj:**  Tumor size T<<C  d-60 survival:  T =90% >> C= 0%.  **CD4/CD8 deletion:**  Abolished MSC-L. effect  **Blocking LIGHT**  3d- before/after, 1 wk, and 3wk MSC-L abolished MSC-L prevention effect | NR | LIGHT.🡪 primes potent immune reaction vs tumour |
| (Abrate et al., 2014) | Solid tumor  Prostate cancer (TRAMPC1/ TRAMPC2) mouse model and  Prostate cancer (TRAMP) mouse model | C57BL/6 and  TRAMPmice  **Solid tumor:**  C= 8  T= 36  **TRAMP**  C= 12  T=13 | C57BL/6 mouse AT-MSC, BM-MSC, Pa= NR  **Solid tumor mouse model:**  C= AT-MSC-IV  T= AT-MSC-CD-IV/ AT-MSC-CD-IV+ 5FC-IP/ BM-MSC-CD-IV+ 5FC-IP/ AT-MSC-CD-IV (2x) + 5FC-IP (1x)/ AT-MSC-CD-IV (2x)+ TAC-IM (2x) + 5FC-IP (1x)  **TRAMP mouse :**  C= (-)/5FC-IP  T= -AT-MSC-CD or BM-MSC-CD -IV+ 5-FC-IP (1 cycle)/ AT-MSC-CD or BM-MSC-CD -IV+ 5-FC-IP (2 cycles) | Retrovirus/ yeast CD-UPRT | **Solid tumor:**  Tumor size:  AT-MSC-CD + TAC (2x) + 5 FC (1x)=  AT-MSC-CD+ 5FC = BM-MSC-CD+ 5FC  < AT-MSC-CD = C  **Prostate Cancer:**  Tumor size:  AT-MSC-CD or BM-MSC-CD +5-FC << C  AT-MSC-CD or BM-MSC-CD + 5-FC (2 cycles) << C | NR | Conversion: 5-FC🡪 5-FU. |
| (Cavarretta et al., 2010) | Solid tumor  Prostate cancer (PC3) mouse model | Nude mouse  **Co-inj PC3 4x10^6^:**  C= 8  T= 20  **Co-inj PC3 3x10^6^**:  C=10  T= 19 | Hu AT-MSC, Pa= NR  **Co-inj PC3 4×10^6^ with:**  C= (-)/ MSC-CDy 40%  T= MSC-CDy 40%+5FC-IP/ MSC-CDy 30%+5FC-IP/ MSC-CDy 20%+5FC-IP/ MSC-CDy 10%+5FC-IP/ MSC-CDy 5%+5FC-IP  **Co-inj PC3 3×10^6^ with:**  C= (-)  T= MSC-CDy 50%+5FC-IP/ MSC-CDy 40%+5FC-IP/ MSC-CDy 10%+5FC- IP/ MSC-CDy 67% IV+5FC-IP/ MSC-CDy 67% IV+5FC-IP/ MSC-CDy 67% IV (2x) + 5FC-IP | Retrovirus/ CD-UPRT | **Co-inj pc3 4X!0^6^**  C: (-)/MSC-CDy 40%: 100% tumour, died d-18/d-12  T: MSC-CDy (20%, 30%, 40%) +5FC: d-16, d-12, d10 - 25% tumour, d-20 regressed  T: MSC-CDy (40%-30%, 10%, 5%) +5-FC - d-40: 100% tumour free  **Co-inj PC3 3× 10^6^:**  100% tumour free: MSC-CDy 50% +5FC  Tumour regression (50%) MSC-CDy 67% IV (2x) + 5FC | NR | Conversion: 5-FC🡪 5-FU. |
| (Zolochevska et al., 2012) | Solid tumor  Prostate cancer (PC3, TC2Ras) mouse model | Balb/c nu/nu  and C57/BL6 mice  **Co-inj PC3:**  C= 9  T= 6  **Co-inj TC2Ras**:C= 6  T= 6 | Hu AT-MSC, Pa= NR  C57/BL6 AT-MSC, Pa= NR  **Co-inj PC3 10^6^ –SC with:**  C= (-)/ hu MSC/ hu MSC-GFP 1:0.2  T= hu MSC-MDA7 1:0.2/ hu MSC- PEDF 1:0.2  **Co-inj TC2Ras – SC with:**  C= (-) /mMSC  T= mMSC- PEDF/ mMSC- MDA-7 | Lentivirus/ MDA-7, PEDF | **Tumour size –co-inj PC3:**  T: hu MSC- PEDF << hu MSC-MDA7, C  d-0-53: hu MSC- PEDF 1:0.2 – no tumour  **tumour size – co-inj TC2Ras:**  mMSC- PEDF << mMSC- MDA7< C  d-0-27: mMSC- PEDF – no tumor | NR | MDA-7= tumour suppressor🡪 induce differentiation, apoptosis, and reduce proliferation. PEDF= inhibitor of angiogenesis, stimulate differentiation and promote apoptosis |
| (Yan et al., 2017) | Solid tumor  Prostate cancer (22Rv1) mouse model | Nude mice  C=10  T= 10 | MSC, Pa= NR  C= PBS IV/MSC.EGFP 1:0.1-IV  T= MSC.scFv-tBid 1: 0.1-IV/ MSC.scFv-Fdt-Bid.1:0.1-IV | Lentivirus/  scFv-Fdt-tBid | Tumor size: d-9 - MSC.scFv-Fdt-tBid.- tumor growth inhibition  Tumor weights: d-30  T MSC.scFv-Fdt-tBid << C < MSC.scFv-tBid  Apoptosis TUNEL: MSC.scFv-Fdt-tBid | NR | scFv-Fdt-tBid= antitumor immuno-pro-apoptotic molecule  Fdt 🡪 cleaved by furin proteases --> transfers from endosome to cytosol 🡪induces cell apoptosis |
| (Lee et al., 2013) | Solid tumor  Prostate cancer (DU145, PC3) mouse model | Nude mice DU145 tumour:  C= 7  T= 14  PC3 tumour:  C=5  T=10 | Hu fetal limbMSC, Pa= NR.  **DU145 tumor:**  C= GCV IP 5x  T= MSC-TK-IV+ GCV- IP 5x/ Dox -IP  **PC3 tumor:**  C= GCV-IP 5x  T= MSC-TK-IV+GCV-IP5x/ Dox-IP | Lentivirus/ SV40-TK | DU145 and PC3 tumor:  Tumor size:  MSC-TK+ GCV, Dox << C  Apoptosis: MSC-TK+ GCV  DU145 tumor:  4/5 - MSC-TK + GCV-🡪 no tumor | NR | SV40-TK 🡪 GCV– phosphorylated🡪 toxic |
| (Zhang et al., 2011) | Solid tumor  Prostate Cancer (PC3) mouse model | Balb/c nu/nu mice  C= 9  T= 3 | Wistar rats BM-MSC  Pa= NR  C= PBS IT/ MSC IT – 3x/ MSC-EGFP-IT – 3x  T= MSC-TT- IT – 3x | Lentivirus/  TNF-α-Tumstatin 45–132 (TT) | d-30 Tumor size:  MSC-TT <<C  proliferating tumor cells : MSC-TT <<C  TUNEL assay:  MSC-TT >>C | NR | TNF-α- 🡪cancer cell apoptosis, inhibited endothelial  cell proliferation  Tumstatin reduce TNF-α systemic toxicity |
| (Wang et al., 2012) | Solid tumor  Prostate cancer (PC3) mouse model | C.B-17/Icr,  (SCID) mice  C= 30  T= 12 | Human BM-MSC, Pa= P4  C= (-)/ MSC AD5 3: 20-IV/MSC 3:20-IV/AD5-IFN-β 3:2x10^4^ viral particles-IV/rec IFN-β (10^5^ IU).-SC  T= MSC IFN-β 3:20-IV/MSC IFN-β 3:2-IV | Adenovirus (AD5)/ IFN-β | Tumor weight:  T= MSC IFN-β 3:20/ MSC IFN-β 3:2 <<C  Survival analysis:  80d: MSC IFN-β 3:20  = MSC IFN-β 3:2 , C= all dead  80-90d: MSC IFN-β 3:20 < MSC IFN-β 3:2 | NR | IFN-β 🡪inhibited malignant cell growth |
| (Chen et al., 2008) | Solid tumor  metastasis melanoma (B16), breast tumor (4T1), and hepatoma (Hca) mouse model | IC57BL/6  mice (B16) / BALB/ c mice (4T1) or  (Hca)  Melanoma/breast tumor/ hepatoma:  C=36 x 3  T= 24 x3 | BALB/c mouse BM-MSC,  Pa= PD7-PD10  Melanoma/breast tumor/ hepatoma:  C=PBS-IV/ MSC-LacZ-IV/MSC-IV  T=free Ad-IL-12-IV, MSC-IL-12 -IV  All injection: 5x every 5d | Adenovirus/ IL-12 | MSC-IL-12 migrated to:  Tumor periphery and capsule after 1 wk  Into tumor after 5 wk  Serous or IT IL-12: MSC-IL-12 > free Ad IL-12  C = no elevated IL-12.  d-20 tumor size:  MSC-IL-12T3 << free Ad-IL-12, C  LN metastases: MSC-IL-12 suppressed and reversed (para-iliac LN 78.8%, axilla LN 100%}, free Ad-IL-12 partly retard, but do not reverse.  Metastasis-lung, liver: MSC-IL-12 (-), free Ad-IL-12, C (+)  d-20-LVD: MSC-IL-12-↓, free Ad-IL-12, C↑ | (-) | IL-12 🡪 rapid destruction of tumor-associated  endothelial cells 🡪 metastasis regression  MSCs homing to tumor or metastatic sites |
| (Kucerova et al., 2008) | Solid tumor  Melanoma  (A375) mouse model | Balb/c nude mice  Co-inj1: C= 11  T= 5  Co-inj2:  C= 20  T= 14  Tumor inj:  C= 16  T= 12 | Hu AT-MSC  Pa=P15-20  **Co-injection A375 SC-1:**  C= (-)/10% MSC-SC+ 5FC-IP  T= 10% MSC-CD-SC + 5FC-IP  **Co-injection A375 SC-2**  C= (-)/ 10% MSC-SC+ 5FC -IP/ 20% MSC-SC+ 5FC-IP  T= 10%MSC-CD-SC + 5FC -IP/ 20%MSC-CD-SC + 5FC -IP  **Tumor- injection**  T= 5FU-IP/ MSC-CD-IV + 5FC-IP  C= MSC-IV/ MSC-CD-IP + 5FC-IP/ Fib-CD-IV+ 5FC-IP | pST2 retrovirus protamine sulfate / Yeast CD-UPRT | **Co-injection-1:**  Tumor size:  Up to d-10: T<< C  d-21: T= C  **co-injection-2:**  Tumor weight d-24:  20% MSC-CD-SC + 5FC-IP <<10% MSC-CD-SC + 5FC-IP  < C  Tumor size:  d-3-7-10-14:  20%MSC-CD-SC + 5FC-IP< 10%MSC-CD-SC + 5FC-IP< C  Tumor free > d14:  20%MSC-CD-SC + 5FC-IP= 89%  20% MSC-SC+ 5FC-IP= 0%  **Tumor injection**:  Tumor size d-23:  MSC-CD-IV + 5FC-IP < 5FU-IP < C | (-) | Conversion: 5-FC🡪 5-FU |
| (Kucerova et al., 2014) | Solid tumor  Melanoma  (A375)  Mouse model | Balb/c-nu/nu  **Co-inj HD**  C=8  T=9  **Co-inj LD**  C= 0  T= 42  **Co-inj AMD-Avastin**  C= 8  T= 16  **Co-inj SU11274**  C= 8  T= 16 | HuAT-MSC, Pa=NR  **Co-injection HD A375 1.5 x10^6^-SC with:**  C= MSC-CD 1:0.2  T= MSC-CD 1:0.2+ 5FC-IP  **Co-injection LD A375/ A375-rel1/A375-rel2 0.5x10^6^-SC with:**  T=A375 + MSC-CD 1:1+5FC-IP/ A375/Rel1 + MSC-CD 1:1+5FC-IP/ A375/Rel2 + MSC-CD 1:1+5FC-IP  **Co-injection A375-rel3 5:1 –AMD-Avastin:**  C= MSC-CD SC+5FC-IP  T= MSC-CD SC+5FC -IP+ AMD SC/ MSC-CD SC+5FC-IP + Avastin-IP  **Co-injection A375-rel3-SU11274:**  C= MSC-CD+5FC-IP  T= MSC-CD+SU11274-IP/ MSC-CD + 5FC-IP+ SU11274-IP | pST2 retrovirus/ CD | **Co-injection HD:**  d-60 tumor free T>C  **co-injection LD:**  d-90 tumor free:  A375> A375-rel1> A375-rel2  **Co-injection A375-rel3- AMD-Avastin:**  Tumor size:  C= T-AMD < T- avastin  Time to >100mm^3^ tumor:  T-AMD < C < T-Avastin  **Co-injection A375-rel3 – SU11274:**  d-60 tumor free  MSC-CD+ 5FC+ SU11274 > MSC-CD+SU11274 | NR | Conversion: 5-FC🡪 5-FU |
| (Tyciakova et al., 2015) | Solid tumor  Melanoma  (A375) mouse model | Balb/c nude mice C=10  T= 8 | HuAT-MSC  Pa= NR  Co-injection A375 SC with:  C=(-)/MSC (9:1)/MSC (4:1)  T=MSC- hTNFα (9:1)/MSC- hTNFα (4:1) | pST40 retrovirus+ protamine sulfate/ hTNFα | d-28 tumor size:  MSC- hTNFα (4:1) < C  d-26 tumor weight:  MSC- hTNFα (9:1)=  MSC- hTNFα (4:1) < C | NR | hTNFα 🡪 apoptosis |
| (Krasikova et al., 2015) | Solid tumor  Melanoma  (B16F10)  Mouse model | C57/BL6 mice  C= 8-10  T= 16-20 | Mouse AT-MSC  Pa=P2 -P 4  B16F10-tumor-injection:  C=(-)  T=MSC-CD-  UPRT IT1x + 5FC–IP-7x/ MSC-CD-UPRT-VP22-IT-1x+5FC-IP–7x | Recombinant plasmid  + electroporation/ CD-UPRT-VP22 | d-20 tumor size :  C >> T  TGI: d-6- d-9: T (>50%) >C  d13: CD-UPRT-VP22 (46%) > CD-UPRT (40%) > C  Survival T> C | NR | Conversion: 5-FC🡪 5-FU  VP22 enhances CD effect on cells |
| (Ahn et al., 2013) | Solid tumor  melanoma  (LMeC) mouse model | Balb/c nude mice  C= 13  T= 8 | Canine AT-MSC  Pa= P4-P6  LMec-tumour injection:  C= PBS-CT-3x/ cis-IP -3x/ MSC- CT-3x  T= MSC-IFN-β CT-3x/ MSC-IFN-β CT+ Cis-IP-3x | Lentiviral vector/ IFN-β | d-30 tumor size:  MSC-IFN-β CT+ Cis < MSC-IFN-β CT <C  d-12- apoptosis:  MSC-IFN-β CT+ Cis > MSC-IFN-β CT > C | NR | IFN-β 🡪 apoptosis, anti-angiogenic |
| (Seo et al., 2011) | Solid tumor  Melanoma  (B16F10)  Mouse  Model | C57BL/6 mice  C=18  T= 6 | Canine AT-MSC  Pa= P5-P8  B16F10-tumor injection:  C=PBS-3x/ Cis IT/ Cis IT+MSC SC 3x  T= Cis IT+ MSC-IFN-β SC 3x | Lentiviral vector/ IFN-β | D17-26-tumor size:  T= Cis-MSC < Cis = PBS  Survival:  T> Cis-MSC > Cis > PBS | (-) | IFN-β 🡪 apoptosis, anti-angiogenic |
| (Grisendi et al., 2015) | Solid tumor  Ewing’s sarcoma  (RD-ES) mouse model | NOD mice (Charles River) C= 14  T=7 | Hu AT-MSC  Pa= up to P12  C= (-)/MSC- IT  T= MSC-TRAIL- IT | Retroviral vector/ TRAIL | Tumor size:  T< MSC= C  Apoptosis:  C< MSC- < T  Angiogenesis:  C= MSC> T | NR | TRAIL 🡪 apoptosis, anti-angiogenesis |
| (You et al., 2009) | Solid tumor  Gastric cancer  (MKN45) mouse model | nude mice  C=14  T=21 | Hu BM-MSC, Pa= P6  C= (-)/ MSC IV + 5FC IV  T= MSC-CD IV + 5-FU IV/ MSC-CD IV + 5-FC IV/ MSC-CD IV + 5-FC) IV x2 | Plasmid + lipofectamine/ CD | Tumor size  d-7, d-9, d-11:  MSC-CD+5FU< MSC-CD+5FC2x < MSC-CD+5FC< C | Reduced BW: MSC-CD- 5FU | Conversion: 5-FC🡪 5-FU |
| (Luetzkendorf et al., 2010) | Solid tumor  Colorectal cancer (CRC: DLD-1, HCT-8, HCT-15, SW480) mouse model | nude mice (Harlan Winkelmann) Co-inj  C=7  T=27  Tumor IV inj  C=3  T=3 | MSC, Pa= up to P12  **Co-injection mixed CRC cells with**:  C=(-)/ MSC SC  T= 20% MSC-TRAIL SC/ 10% MSC-TRAIL SC/ 3% MSC-TRAIL SC/ 1% MSC-TRAIL SC  **DLD-1 tumor IV injection: d-2-4-7-15:**  C=PBS/20% MSC IV  T= 20% MSC-TRAIL IV | Lentiviral vector/ TRAIL | **Co-injection**:  d-17 tumor size:  20% MSC-TRAIL << 10% MSC-TRAIL < C= 3% MSC-TRAIL = 1% MSC-TRAIL  **Tumor IV injection**  d-18 tumour size:  T= C | (-) | TRAIL 🡪 apoptosis |
| (Gao et al., 2010) | Solid tumor  Renal cancer  (786-0 RCC) mouse model | nude mice  C= 40  T= 40 | Hu BM-MSC  Pa=P3 or P4  C=(-)/MSC (1:0.1)IV  T= MSC-IL12 (1:0.1) IV/ MSC-IL12 (1:0.05) IV | Adenovirus/ IL-12 | d-32 tumor size:  T- HD < T- LD < C  Survival  T- HD > T- LD >C  IT, serum IL12, IFN-γ: T > C | NR | IL-12 activate T cells, NK cells 🡪 IFN-γ |
| (Yan et al., 2013) | Solid tumor  Non-Hodgkin’s Lymphoma  (BJAB) mouse model | NOD/SCID mice  C= 20  T= 10 | Hu UC-MSC  Pa=P3-P5  C= PBS/MSC IV/ MSC Con IV/ MSC-scFvCD20 IV  T= MSC.ISZ-sTRAIL IV/ MSC-scFvCD20-sTRAIL IV | Lentivirus/ scFvCD20-sTRAIL | d-24 tumor size:  MSC-scFvCD20-sTRAIL = MSC.ISZ-sTRAIL < C  d-24 tumor weight  C >> MSC.ISZ-sTRAIL > MSC-scFvCD20-sTRAIL | NR | TRAIL 🡪 apoptosis  scFvCD20 🡪 antibody against CD20  BJAB cells are CD20 (+) |
| (Yao et al., 2017) | Metastatic lung cancer  (4T1) mouse model | babl-c mice  C= 22  T= 33 | SD Rat BM-MSC  Pa= P2-P8  **IV:** d- 6-9-12-15-18:  C= saline/ MSC  T=free dox/ BPCD/ MSC-BPCD | Biotinilated MSCs/ avidin-BPCD | 24-h bio-distribution  MSC-BPCD vs MSC: lung: 93 vs 85.5%, liver: 6.3 vs 7%,  Spleen: 0.7 vs 7.5%.  MSC - lung - 2 wk.  ILS: MSC-BPCD (+26.6%) > BPCD (+58.5%) > free dox Apoptosis: MSC-BPCD> BPCD > free dox> saline> MSC | NR | MSCs as vehicle for dox delivery |
| (Matuskova et al., 2015) | Metastatic lung cancer  (MDA-MB-321) mouse model | SCID/bg mice  C= 26  T= 22 | Human AT-MSC  Pa= NR  C: (-)/ MSC-IV  T: MSC-CD-IV+ 5FC-IP-14d/ MSC-TK IV+ GCV-IP-14d/ MSC-CD+ MSC-TK 1:1 -IV+ 5FC+ GCV-IP-14d  (all 5FC, GCV- 2 cycles) | pST2, pAPtk retrovirus / Yeast CD-UPRT, HSV-TK | d-40 MDA-MB-321 –in lungs:  (-)=11/15  MSC =9/11  MSC-CD+ 5FC =4/4  MSC-TK+ GCV =3/6  MSC-CD+ MSC-TK 1:1+5FC+GCV =0/12 | NR | Synergistic cytotoxicity from Conversion: 5-FC🡪 5-FU and HSV-TK |
| (Hu et al., 2014) | Solid tumor and metastatic lung tumor  (B16BL6) mouse model | C57BL6 mice  Metastasis  C=12  T=36  Solid tumor  C= 12  T=54  Survival:  C= 20  T= 60 | SD Rat BM-MSC  Pa= P2-P6  Metastasis -IV injection  C=(-)/PBS  T=MSC(10^2^)/ MSC (10^4^)/ MSC (10^6^)/ SP-IL12/ MSC IL12 (10^6^)  Solid tumor –injection🡪 tumor size, survival  C2= (-)/ PBS-IV  T=MSC (10^4^) IV/ MSC (10^5^) IV/ MSC (10^6^) IV/ SP-IL12 IV/MSC-IL12 (10^6^) IV/ MSC (10^6^) IT/ SP-IL12 IT/ MSC-IL12 (10^6^) IT | Spermin-Pullulan-DNA nanoparticles/ IL-12 gene | **Metastasis:**  d14-metastasis no:  MSC (10^6^) > (10^4^)= (10^2^)= C(-)  D21-metastasis no:  MSC IL12 < SP-IL12 << PBS  **Solid tumor:**  IV-tumor size: C(-)> MSC 10^6^=10^5^>10^4^ (  IV-tumor size: PBS> MSC-IL12> SP-IL12 (NS)  IT tumor size: MSC-IL12= SP-IL12<< PBS= MSC(10^6^)  d-30: Survival IV- IT:  MSC-IL12> SP-IL12 > PBS> MSC (10^6^) | NR | IL-12 🡪 cytotoxic NK activity↑  cytolytic T cell↑  interferon-γ ↑.  MSC-IL12 >< MSC tumor growth effect |
| (Xin et al., 2009) | Multiple lung metastasis  (C26, LLC)  Mouse model | BALB/c, C57BL/6 mice  C=48  T= 32 | BALB/c, C57BL/6 mouse BM-MSC, Pa= P3-P5  BALB/c–C26, C57BL/6-LLC  C= PBS- ITr/ MSC-LacZ-ITr/ Fib-FKN -ITr  T= MSC-FKN- ITr/ Ad-FKN -ITr | Adenovirus vector/ CX3CL1 (Fractalkine) | d12-metastasis no:  BALB/c –C26: MSC-FKN < Ad-FKN << C  C57BL/6-LLC: MSC-FKN << Ad-FKN = C  survival:  BALB/c –C26:  MSC-FKN (33d) >> Ad-FKN, C (23-19d)  C57BL/6-LLC:  MSC-FKN (17d) >> Ad-FKN, C (15-10d) | Ad-FKN🡪 Immune reaction | MSC- homing  CX3CL1 🡪 antitumor  effect through NK cells and T lymphoc  ytes. |
| (Ren et al., 2008) | Lung metastasis  (TRAMPC2)  Mouse model. | C57BL/6 mice C=30  T= 20 | C57BL/6 mice BM-MSC  Pa= P4-P8  C= (-)/ MSC-IV 2x/ MSC- GFP-IV -2x  T= MSC p IFN-IV-2x/ MSC-rAAV IFN-IV-2x | rAAV/ IFN-β | d75- metastasis/ weight: MSC-rAAV-IFN< MSC p-IFN = C  d-75-blood vessels:  MSC-rAAV-IFN<< C  d-75 apoptosis:  MSC-rAAV-IFN>> C | NR | IFN-β suppresses tumor cell growth  by induction of differentiation, S-phase accumulation and apoptosis. |
| (Conrad et al., 2011) | Orthotopic pancreatic carcinoma (panc02), and mammary adenoca  mouse model | C57BL/6 mice,  Balb-neuT (*neuT*+*/neuT*^-^) **Panc02:**  C= 27  T= 7  **Breast Ca**  C= NR  T= NR | C57BL/6 homozygous  *p53^-^* mouse BM-MSC  PA= NR  **C57BL/6-Panc02**:  C= PBS-IV/GCV-IV d-5-6-7/ p53^−^MSC-RFP-IV/ week 3x  T= (*p53*^−^MSC-TK - IV+GCV IV d-5-6-7 ASC)/week -3x  **Breast ca :**  C= PBS-IV/ *p53*^−^MSC-*RFP-*IV/week 3x  T= (*p53*^−^MSC-TK IV+ GCV IV d-5-6-7-8 ASC)/week -3x | CMV vector/ *Tie2-RFP-HSV-TK* gene | **Pancreatic cancer**  Tumor weight:  MSC-TK-GCV<< MSC-RFP< PBS< GCV  Tumor size:  T<< PBS  **Breasr cancer:**  Tumor weight~body weight: T<< C  d-6-survival:  T>> PBS > MSC-RFP | NR | HSV-TK 🡪 GCV– phosphorylated🡪 toxic  Tie🡪 homing to tumor blood vessels |
| (Mirzaei et al., 2018) | Lung metastasis (B16F10) mouse model | C57BL/6 mice  C= 80  T= 20 | Hu AT-MSC, Pa=P3  C= (-)/PBS IV/MSC IV/ MSC-mock IV  T= MSC-CXCL10 IV | Plasmid-lipofectamine/ CXCL10 | Median survival time:  T (50d) >> MSC (26d) = PBS (26d) > MSC mock (24d)> C(-) (23d)  Apoptosis, activated T cell no: T>> C  Mean vessel density, T reg cell no: T<< C | (-) | CXCL10 🡪 apoptosis, anti-angiogenic on various cancer cells |
| (Kim et al., 2013) | Lung metastasis  (Renca) mouse model | Balb/mice  **Tr d1-d**7  C=22  T= 66  Survival= 5 x 9  **Multiple inj**  C= 6  T=38  Lung nodule= 3 x 3 | Rat BM-MSC, Pa= P5-P6  **Tr d1, d7 after Renca IV:**  C= PBS IV+d2-GCV-IP 7d/ 5x10^5^ MSC-GFP IV + d2-GCV-IP 7d  T= 5x10^5^ MSC-TK IV + d2 GCV-IP 7d/ 5x10^5^ MSC-dTRAIL IV + d2 GCV-IP 7d/ 5x10^5^ MSC-TRAIL-TK- IV + d2 GCV-IP 7d  **d7- various dose- multiple inj + d2 GCV-IP-7d:**  C= 5x10^5^ MSC-GFP IV  T= 5x10^5^ MSC-TRAIL-TK IV-1x/ 10^6^ MSC-TRAIL-TK IV-1x/ 1.5x10^6^ MSC-TRAIL-TK IV-1x/ 5x10^5^ MSC-TRAIL-TK IV -2x/ 5x10^5^ MSC-TRAIL-TK IV -2x | Adenovirus/ TRAIL-TK | Tr d1, d7: d14 lung nodule no: MSC-TRAIL-TK < MSC-TRAIL= MSC-TK << MSC  Tr d7: d100 survival: MSC -TRAIL-TK > MSC-TK > MSC-TRAIL > C  **d7- various dose multiple injection:**  d120-survival rate:  T-LD-3x (=100%) >> T-LD-2x >T-HD=T-MD> T-LD>> C  d-60 lung nodules no: T-LD 1x >> T-LD 2x > T-LD 3x (=0) | NR | TRAIL 🡪 apoptosis  TK 🡪 conversion of nontoxic GCV into toxic form |
| (Harati et al., 2015) | Liver metastasis  (SW48)  mouse model | C57BL/6 mice Non Me C= 12  C= 18  T= 6 | huBM-MSC  Pa=NR  Non Me C=PBS IV/ MSC IV  C= PBS IV/ MSC IV/ MSC-GFP IV  T= MSC-Lcn2 IV | pEGFPN1 plasmid + FuGENE transfection/ Lipocalin-2 | Liver metastasis  Non Me C (0/6, 0/5)< T(1/5) << C (3/4-4/5)  VEGF expression  Non Me C< T< C | NR | Lipocalin-2🡪Inhibit HIF-1a, FA-kinase phosphorylation, VEGF synthesis |
| (Lakota et al., 2015) | Lung metastasis- tongue squamous ca  T2N0M0  Surgery 2x | Human, male 41 y- case report  T= 1 | AT-MSC, NR  d-1-7: 2.5 g 5FC/250 ml solution –IV-2x/d  d-2: 60x10^6^MSC-IV | pST2 retrovirus + protamine sulfate/ CD-UPRT | d-6: CT scan- no difference – lung metastasis  d-40: CT scan: progression of metastases | Fever, resolved after antipyretic, thrombocytopenia, neutropenia | Conversion: 5-FC🡪 5-FU |

MSC= mesenchymal stem cell, C= control, T= treatment, hu= h= human, BM-MSC= human bone marrow mesenchymal cells, Pa= passage, NR= not reported, PBS= Phosphate buffered saline, 5FC= 5fluorocytosine, 5FU=5fluorouracil, PSGL-1= P-selectin glycoprotein ligand-1, SLEX= Sialyl-Lewis X= homing factors, CD= cytosine deaminase, OPG= osteoprotegerin (therapeutic factors), c3-MSC= Murine C3H10T1/2 MSC line, PC3= androgen-independent hu prostate ca cell line from a bone metastasis, ATF= urokinase-type plasminogen antagonist amino-terminal fragment, d- =day-, GBM8= glioblastoma multiforme cells, IC= Intra cerebral, s-TRAIL= secreted TRAIL, TRAIL= TNF related apoptosis-inducing ligand, TNF= tumor necrosis factor, Act-Cas3= Activated caspase-3(+) cells, AT-MSC= Adipose tissue derived MSC, IP= Intraperitoneal, Ive= Intra ventricular, CL= contralateral, C6= rat glio(blasto)ma cell line, CD-UPRT= cytosine deaminase::uracil Phospho-Ribosyl-Transferase, R= tumour resection, IVeP= Intraventricular Pump, AT-CD-IC= AT-MSC-CD intracerebral, AT-CD-P= AT-MSC-CD intracerebral pump, 9L= rat glioma cells, SC= subcutaneous, SD= Spraque Dawley, U87MG= human glioma cell line, Ki67= proliferation marker, ATS= at tumor site, ACLS= at contra lateral site, ESC= embryonic stem cells, rec= recombinant, Bacvec= insect baculovirus vector (plasmid pFastBac1) , Lenvec= lentiviral vector/HSV-TK, GCV= Ganciclovir, HSV-TK= herpes simplex virus thymidine kinase, Gli36vIII= human glioblastoma mutiforme cells expressing a constitutively active variant of Epidermal growth factor receptor (EGFR), MTX= methotrexate, PEG= poly ethylene glycol, NC= nanocomposite, ET= edge of tumor, PL=peptide ligand, IL13Ra2=Interleukin13 Receptora2, g= gram, Cal72= hu osteosarcoma cell line, HT-29= Human colon adenocarcinoma cell line, inj= injection, T-LD= Treatment low dose, T-HD= treatment high dose, huH7= Human hepato cellular carcinoma cell line, CMV= cytomegalovirus, NIS= sodium Iodide transporter, SKOV3= ovarian ca cells, NTR= nitroreductase, IT= Intra tumour, TC1 cells= murine lung epithelial-cell line, LLC= Lewis lung carcinoma cells, VP22= Herpes Simplex VirusType1 tegument protein, A2780= Human ovarian adenocarcinoma cell line, TUBO= murine breast tumor cell line, LIGHT= homologous to lymphotoxin, LTßR-Ig= lymphotoxin ß receptor Imunnoglobulin, TRAMP= transgenic adenocarcinoma of the mouse prostate, TRAMPC1/TRAMPC2= murine prostate tumour cells, TAC= Tacrolimus (immunosuppresant), TC2Ras= TRAMPC2-Ras, MDA-7= IL 24= Melanoma differentiation associated gene-7, PEDF= pigment epithelium-derived factor, 22Rv1= Human prostate cancer cell line, scFv-Fdt-tBid= single chain anti-g-SM antibody (scFv)-short furin cleavage sequence from diphtheria toxin (Fdt)-activated truncated Bid (tBid), DU145= human prostate cancer cell, dox= doxorubicin, TT= TNF-a-Tumstatin 45–132 fusion protein, EGFP= enhanced green fluorescent protein, SCID= severe combined immune deficiency, B16= melanoma cells, 4T1= breast tumor cells, Hca= Hepatoma cells, PD= population doubling, Ad= adenovirus, wk= week(s), LN= lymph node, A375= human melanoma cells, rel(1-3)= relapse 1-3x tumour cells, AMD= AMD3100= SDF-1a/CXCR4 signaling inhibitor, SU1127= small molecule tyrosine kinase inhibitor of c-Met signaling, B16F10= murine melanoma cells, TGI= tumor growth inhibition, LMeC= Canine melanoma cells, CT= circum-tumoral, Cis= Cisplatin, RD-ES= Ewing's sarcoma cell line, MKN45= human gastric cancer cells, CRC= colorectal cancer, DLD-1, HCT-8, HCT-15, SW-480= human colorectal cancer cell lines, 786-0 RCC= human renal cancer cell line, BJAB= CD 20 positive B-cell lymphoma line, scFv= single chain Fv antibody fragment, scFvCD20= CD20-specific scFv, Con= CopGFP (vector control), ISZ= isoleucine zipper, PPCD= PEG PAMAM-cis-aconityl-DOX, BPCD= Biotinylated PPCD, h= hour(s), ILS= increased life span, DA-MB231 cells= hu breast ductal adenocarcinoma cell line, B16BL6= melanoma cells, SP= spermin pululan, no= number, NS= not significant, C26= Murine colon adenocarcinoma cell line, ITr= Intra trachea, Fib= fibroblast, FKN= fractalkine (CX3CL1), IFN= interferon, p-IFN= plasmid-IFN, Panc02= mouse pancreatic cancer cells, adenoca= adenocarcinoma, Balb-neuT (neuT+/neuT-) mouse= transgenic mice with spontaneous focal mammary adenoca, RFP= red fluorescent protein, ASC= after stem cell, Tie2-RFP-HSV-TK gene= RFP + angiopoietin receptor tyrosine kinase, CXCL10= interferon-inducible protein (IP-10) that possesses various anti-cancer effects, Renca= murine renal carcinoma cell line, T-MD= treatment medium dose, SW48= human colon cancer cell line, non Me= non metastasis, Lcn2= lipocalin-2, HIF= hypoxia inducible factor, VEGF= vascular endothelial growth factor
