# Supplementary material for: Enhancement of the Therapeutic Capacity of Mesenchymal Stem Cells by Genetic Modification: A Systematic Review
Source: Front Cell Dev Biol. 2020 Oct 30;8:587776. doi: 10.3389/fcell.2020.587776 (PMC7661472; doi:10.3389/fcell.2020.587776)
Supplement: Supplementary file 2 [file Table_2.docx]

**Table S2. Details of studies using genetically engineered MSCs for treating non-cancer conditions**

| Reference | treated disease/disease models | Species/number of subjects | MSC source and passage, treatments | Vector/gene-expressed prot-drug | outcome | adverse event | mechanism of action |
| --- | --- | --- | --- | --- | --- | --- | --- |
| (Qi et al., 2015) | Liver Transplanta-tion (Allograft)  mouse model | LEW, BN, ACI Rats  C= 6  T= 46 | LEW, BN, ACI Rat BM-MSC  Pa: P6-P8  LEW= liver donor  BN = liver donor  ACI= recipient  **After liver transplant:**  C: (-)  T: LEW MSC-PV/ LEW MSC-Foxp3-PV/ LEW MSC-Vector-PV/ LEW MSC-Foxp3- PV+ RAM CD25 mAb-IP d-2, 0,+2/ LEW MSC-Foxp3 PV + rat IgG/ BN MSC-Foxp3-PV/ ACI MSC-Foxp3-PV | Lentivirus/ Foxp3 | Median survival time:  LEW MSC-Foxp3= LEW MSC-Foxp3+IgG (100d) > ACI MSC-Foxp3 (57d)> BN MSC-Foxp3 (25.5d)> LEW MSC (21d)= LEW MSC-Vector (20d) > LEW MSC-Foxp3 + CD25 mAb (14d)=C (-) (13.5d)  d-7- RAI :  C(-)> LEW MSC = LEW MSC-Vector> LEW MSC-Foxp3  d-7 ALT, AST, TBIL:  C(-)> LEW MSC = LEW MSC-Vector> LEW MSC-Foxp3 | NR | Foxp3:  Cell-cell contact mechanism (PD-L1↑), Treg↑🡪Immunomodulatory |
| (Niu et al., 2014) | Liver Transplanta-tion (Allograft) mouse model | DA, LEW Rats  C: 14  T: 42 | DA Rat BM-MSC  Pa: P8-P11  DA= liver donor  LEW= recipient  30 min after liver transplant – RJV:  C: saline  T: MSC/ MSC-vector/ MSC-IL-10 | Lentivirus/ IL-10 | Median survival time:  MSC-IL-10>MSC-vector = MSC> C  RAI score:  C> MSC-IL-10= MSC-vector = MSC | NR | IL-10🡪Th17, IL-17, IL-23, IL-6, IFN-γ, TNF-α↓  TReg, IL-10, TGF-β1↑ |
| (Fransson et al., 2014) | Experimental autoimmune encephalo-myelitis (MOG_35-55_ peptide SC immunization) mouse model | C57BL/6 mice C=10  T= 20 | Mouse BM-MSC  Pa: P5-P8  IN, IP delivery,:  C: PBS/ MSC mock  T: MSC-CARα-MOG  IN cured animal-2^nd^ challenge | Lentivirus/ CARα-MOG | IN- EAE score :  d17: PBS= MSC mock= T  d23: PBS> MSC mock= T  d-27-30: PBS >MSC mock> T (symptom free)  IP-EAE score:  d17-23: PBS= MSC mock= T  d25-30: PBS= MSC mock> T  2^nd^ challenge-EAE score:  d1-20: MSC mock>T | NR | MOG🡪 axons damage ↑  CARα-MOG 🡪 binds MOG  MSC CARαMOG & MSC mock:  CD8^+^IFN-γ^+^ & CD4^+^IFN-γ^+^ 🡪 low  CD4^+^IL17^+^ 🡪 high  IL12↓ IFN-γ↓ |
| (Wang et al., 2015 b) | Nerve injury  (mandibular distraction) rabbit model | New Zealand white rabbits  C: 10  T: 10 | Rabbit allo BM-MSC  Pa: P2  C: MSC-vector  T: MSC- hNGFβ | Lentivirus/ hNGFβ | Nerve fibers: T> C  Myelin debris: T< C  Myelinated fiber density: T> C | NR | NGF🡪 nerve fiber regeneration |
| (Nakamura et al., 2013) | Wound healing 15-mm full-thickness skin defects rat model | Fisher 344 rats C= 6  T= 12 | Rat BM-MSC  Pa: P2-P3  C: PBS  T: MSC/ MSC-SDF-1 | Spermine-pullulan/ SDF-1 | Wound closure:  d5:MSC-SDF1>MSC=C  d10-15: MSC-SDF-1> MSC> C  Neo-epithelium length:  MSC-SDF1= MSC> C  Blood vessel no:  MSC-SDF1> MSC> C | NR | SDF-1🡪 VEGF, HGF, IL-6↑  🡪dermal fibroblast migration↑ |
| (Beegle et al., 2016) | Critical limb ischemia mouse model | NSG, B2M mice C: 24  T: 12-24 | huBM-MSC  Pa: P3-P6  C: NS/ MSC+ Axt/ MSC-VEGF+ Axt  T: MSC/ MSC-VEGF | Lentivirus/ VEGF | Wound closure-scratch assay: MSC-VEGF> MSC> MSC+ Axt= MSC-VEGF+ Axt  Blood flow:  wk-0-5: NS= MSC= MSC-VEGF  wk-6-9: NS< MSC= MSC-VEGF  wk-10: NS= MSC< MSC-VEGF | No hemangioma | VEGF 🡪 angiogenesis. |
| (Roudkenar et al., 2018) | Acute kidney injury (cisplatin IP) mouse model | SD rats Normal rat:  C: 36  T: 12  AKI rat  C= 36  T= 12 | Rat BM-MSC  Pa: NR  Normal rat:  C: saline/ MSC/ MSC-Vector  T: MSC-Lcn2  AKI rat  C: (-)/ MSC/ MSC-Vector  T: MSC-Lcn2 | pcDNA3.1/CT-GFP-plasmid/ Lcn2 | Cast no, tubular nec:  AKI C(-) >MSC= MSC vector> AKI-T > Nr-C  sCrea- BUN - d4:  Nr C=Nr-T< AKI-C(-)= AKI-MSC= AKI-MSC-vector= AKI-T  sCrea - d21:  Nr-C= Nr-T= AKI-T< AKI-MSC= AKI-MSC vector< AKI C(-)  BUN –d21:  Nr-C= Nr-T< AKI-T< AKI-MSC= AKI-MSC vector< AKI C(-) | NR | Lcn2 🡪  GF↑ = Repair marker↑  Injury marker↓ |
| (Zhu et al., 2012) | Infarct Myocardium mouse model | C57/BL6 mice  C= 10  T= 10 | Mice BM-MSC  Pa: <P5  Intramyocardial Inj:  Non Infarc:  C= MSC-VEGF  Infarct mice:  C= DMEM  T: MSC/ MSC-VEGF | hPAMAM/ VEGF | d-3 intramyocard VEGF  C< MSC< MSC-VEGF  LVEF, LVFS, LVAWTed, LVATWTes:  DMEM< MSC< MSC-VEGF< non infarct MSC-VEGF  LVIDed, LVIDes:  DMEM> MSC> MSC-VEGF> non infartct MSC-VEGF  Capillary count:  DMEM< non infarct MSC-VEGF< MSC< MSC-VEGF | NR | VEGF 🡪 angiogenesis  ↑heart function↑ |
| (Zhang et al., 2010) | Infarct Myocardium rat model  . | SD rats C= 12  T= 30 | Rat BM-MSC  Pa: P2-P4  Myocard infarct –cell sheet implant:  C: (-)  T: MSC+NS/ MSC-CXCR4 +NS/ MSC +Dip-IV 2x/d/ MSC-CXCR4+ Dip-IV 2x/d/ MSC-siRNA+ Dip-IV 2x/d | Adenovirus/ CXCR4 | Myocardial DPP-IV:  d7: C= MSC+NS> MSC+ Dip  d28: C= MSC+NS= MSC +Dip  Capillary density in cell sheet: MSC+NS= MSC+ Dip= MSC-siRNA+ Dip< MSC-CXCR4+NS< MSC-CXCR4+Dip  Capillary density in border zone:  C< MSC+NS= MSC+Dip= MSC-siRNA+ Dip< MSC-CXCR4+NS< MSC-CXCR4+Dip  Cell engraftment:  MSC+NS= MSC+Dip= MSC-siRNA+Dip< MSC-CXCR4+NS< MSC-CXCR4+Dip  Apoptotic cells:  C= MSC+NS> MSC+Dip  Fibrotic area:  C> MSC+NS= MSC-siRNA+ Dip> MSC-CXCR4+NS= MSC+ Dip> MSC-CXCR4+Dip  LVDd, LVDs:  C> MSC+NS= MSC+ Dip= siRNA+ Dip> MSC-CXCR4+NS> MSC-CXCR4+ Dip  Ejection fraction, Fractional shortening:  d1-wk1: C= T  wk2-4: MSC-CXCR4+Dip> MSC-CXCR4+NS> MSC+Dip> MSC+NS= MSC-siRNA+ Dip> C(-)  Anterior wall thickness:  MSC-CXCR4+NS< MSC-CXCR4+Dip | NR | SDF-1α binds to CXCR4 and is cleaved by DPP-IV  CXCR4 + Dip :  Apoptosis↓, Angiogenesis↑, Cell engraftment↑  heart function↑ |
| (Huang et al., 2010) | Infarct Myocardium rat model  . | SD rats Sham= 6  C= 12  T= 18 | Rat BM-MSC  Pa: P2-P4  C: Sham/ MI/ MI-P  T: MSC-P/ MSC-CXCR4-P/ MSC-siRNA-P | Adenovirus/ CXCR4 | LVDd:  Sham< MSC-CXCR4-P < MSC-P= C= MSC-siRNA-P  LVDs:  sham< MSC-CXCR4-P < MSC-P< C= MSC-siRNA-P  LVFS, LVEF:  sham> MSC-CXCR4-P> MSC-P >C= MSC-siRNA-P  Cell engraftment:  MSC-P= MSC-siRNA-P < MSC-CXCR4-P  Capillary Density:  C= MSC-siRNA-P< MSC-P< MSC-CXCR4-P  Fibrotic area:  C> MSC-siRNA-P >MCC-P= MSC-CXCR4-P  Anterior wall thickness:  C= MSC-siRNA-P< MSC-P< MSC-CXCR4-P  Ejection fraction, fractional shortening:  Sham< MSC-CXCR4-P < MSC-P= C= MSC-siRNA-P | NR | CXCR4🡪  Myocardial remodelling↓, Angiogenesis↑, Cell engraftment↑ heart function↑ |
| (Li et al., 2007) | Infarct Myocardium rat model | LEW rats C= 12  T= 18 | Rat BM-MSC  Pa: P3  C: Sham/ MI+ DMEM  T: MSC/ MSC-Vector/ MSC-Bcl-2 | jetPEI/ Bcl-2 | In vitro hypoxia VEGF secretion:  MSC-Bcl-2> MSC-Vector= MSC  Cell engraftment:  d4-wk3-wk6: MSC-Bcl-2> MSC-Vector  Blood Vessel Density:  C< MSC-Vector< MSC-Bcl-2  Scar Size:  C> MSC-Vector> MSC-Bcl-2  Heart function (+dP/dt, -(-dP/dt)):  sham> MSC-Bcl-2> MSC-Vector >C | No tumor, no diff into bone, adipose, cartilage | MSC-Bcl-2🡪 VEGF↑  Angiogenesis↑ Cell engraftment↑ heart function↑ |
| (Kumar and Ponnazhagan, 2007) | Normal immune-competent mouse –model | C57BL/6 mice  C= 24  T= 8 | Murine C57BL/6-Tg (ACTbEGF)1Osb/J- allo BM-MSCs  Pa= P4-P8  C=PBS-IV/ MSC-IV/ MSC-GFP-IV  T=MSC- integrin-IV | Adeno-associated virus/ GFP, ɑ4-integrin | Percent engraftment: T> MSC-GFP | NR | ɑ4β1 integrin 🡪 mediate initial capture, rolling & firm attachment to one marrow and endothelial cells 🡪 improve homing |
| (Chang et al., 2004) | Cranial bone defect rabbit model | New zealand white rabbit  T=15 | Rabbit allo BM-MSCs  Pa=NR  On site implantation:  T= Alg +MSC-βgal- (left)/ Alg + MSC-BMP2 (Right) | Adenovirus/ βgal,  hBMP2 | Cranial bone formation (3-mo): Right> Left | NR | BMP2 🡪 🡪osteogenesis |
| (Chang et al., 2010b) | Cranial bone defect pig model | Mitsae Pig T= 40 | Pig auto BM-MSCs  Pa: NR  On site implantation  T=MSC-BMP-UPAlg (Right)/ MSC-Gal-UPAlg (Left)/ MSC-BMP-Alg (Right)/ MSC-Gal-Alg (Left)/ MSC-BMP-Alg-RGD (Right)/ MSC-Gal-Alg-RGD (Left)/ MSC-BMP-Col (Right)/ MSC-Gal-Col (Left) | Adenovirus/ βgal,  hBMP2 | Cranial bone formation (3-mo): MSC-BMP-Col > MSC-BMP-UPAlg > MSC-BMP-Alg-RGD > MSC-BMP-Alg  Right > Left | NR | BMP2 🡪 osteogenesis  RGD 🡪inhibits initial chondrogenesis  Col 🡪degradation faster 🡪Improve bone regeneration |
| (Tsuchida et al., 2003) | Femoral segmental bone defect rat model | Fischer 344 (RT1) rats (Syn),  BN rats (Allo)  T= 42 | Fischer 344 (RT1) BM-MSCs  Pa: P2  On site implantation  T=MSC-BMP-Syn/ MSC-βGal-Syn/ MSC -BMP-Allo/ MSC-βGal-Allo/ MSC-BMP + FK 506- Allo/ MSC-βGal + FK 506 -Allo | Adenovirus/ βgal, hBMP2 | Bone formation:  MSC-BMP2 + FK 506- Allo = MSC-BMP2-Syn > MSC-βGal-Syn  MSC-BMP2 + FK 506- Allo >MSC--BMP2-Allo> MSC-βGal-Allo = MSC-βGal + FK 506 -Allo | Weight loss except FK506 | BMP2🡪osteogenesis |
| (Lin et al., 2015) | Femoral bone defect minipig model | Taiwan Lee-Sung minipigs C=4  T=5 | Pig allo ASCs  Pa: P3-P5  On site implantation  C=ASC- Mock  T= ASC- BMP-VEGF | Baculovirus/ hBMP2, hVEGF | Bone stiffness (12weeks)  NOS> T> C | NR | BMP2 🡪promote bone formation  VEGF 🡪vascular network |
| (Lin et al., 2012b) | Femoral bone defect rabbit model | New Zealand White rabbits  C= 4  T= 11 | Rabbit allo ASCs  Pa: P3-P5  On site implantation  C=ASC-Mock  T= ASC-BMP + ASC -VEGF-tr/ ASC-BMP +ASC-VEGF-per | Baculovirus/ hBMP2, hVEGF | Bone healing (bone volume, density, mechanical properties) anginogenesis:  ASC-BMP + ASC-VEGF-per > ASC-BMP + ASC-VEGF-tr > ASC-mock | NR | BMP2 🡪promote bone formation  VEGF 🡪vascular network |
| (Lin et al., 2010) | Femoral bone defect rabbit model | New Zealand White rabbits  C= 45  T= 141 | Rabbit allo BM-MSC  Pa= P4  On site implantation: PLGA and:  C= (-)/ MSC-Mock  T= MSC BMP-MOI-50/ MSC-BMP-MOI-50+ MSC-VEGF-MOI-150- 1:4/ MSC-BMP-MOI-100+ MSC-VEGF-MOI-150- 1:4 | Baculovirus/ hBMP2, hVEGF | Bone healing:  Wk 2-4: C= MSC-BMP-MOI-50< MSC-BMP-MOI-50+ MSC-VEGF-MOI-150-1:4< MSC-BMP-MOI-100+ MSC-VEGF-MOI-150- 1:4  Wk 8: C< MSC-BMP-MOI-50< MSC-BMP-MOI-50+ MSC-VEGF-MOI-150-1:4= MSC-BMP-MOI-100+ MSC-VEGF-MOI-150- 1:4  Wk8: mechanical property  C< MSC-BMP-MOI-50< MSC-BMP-MOI-50+ MSC-VEGF-MOI-150-1:4< MSC-BMP-MOI-100+ MSC-VEGF-MOI-150- 1:4< NOS  wk-8: angiogenesis: C< MSC-BMP-MOI-50< MSC-BMP-MOI-50+ MSC-VEGF-MOI-150-1:4< MSC-BMP-MOI-100+ MSC-VEGF-MOI-150- 1:4 | NR | BMP2 🡪 bone formation  VEGF🡪 angiogenesis |
| (Lin et al., 2012a) | Critical-size calvarial bone defect rabit model | New Zealand White rabbits  C= 3  T= 10 | Rabbit allo BM-MSC  Pa: P3-P4  On site implantation: PLGA only (left), cell + PLGA (right)  C= MSC-mock  T= MSC-BMP + MSC-VEGF-tr/ MSC-BMP + MSC-VEGF-per | Baculovirus/ hBMP2, hVEGF | wk-12-bone regeneration  NOS> MSC-BMP + MSC-VEGF-per> MSC-BMP + MSC-VEGF-tr> C  PLGA scaffold improve bone regeneration in T | NR | BMP2 🡪 bone formation  VEGF🡪 angiogenesis |
| (Lin et al., 2013) | Calvarial bone defect rabbit model | New Zealand White rabbits  C= 3  T= 32 | Rabbit allo ASCs  Pa: P3-P5  On site implantation:  Gelatin+cells (left), PLGA+cells (right)  C= MSC-mock  T= MSC-BMP/ MSC-TGF | Baculovirus/ hBMP2, TGF-β3 | Bone formation -volume –density- wk 2-4-12*:  MSC-BMP > MSC-TGF,  Left> Right  Relative bone metabolic activity -wk-2-4-12:  C: Left= Right  T: Left> Right  wk-2: MSC-BMP-Left>> MSC-TGF-Left > C-left | NR | BMP2 🡪 bone formation  TGF- β3🡪 chondro-inductive activity  PLGA promotes soft tissue formation  Gelatin promotes bone formation via endochondral ossification pathway |
| (Zhao et al., 2010) | Critical size mandibular full thickness defect rat model | Fisher 344 rats  C= 16  T= 6 | Rat BM-SCs  Pa: P3  β-TCP implant and:  C= (-)/ MSC/ MSC-GFP  T= MSC-BMP | Adenovirus/ hBMP2 | Wk-8-new bone area:  T> MSC-GFP= MSC> C(-)  wk-8-fibrous connective tissue: T< C  wk-8-residual scaffold: T> C | NR | β-TCP scaffold 🡪 facilitates vascular invasion and bone development  BMP2 🡪 elevated mineralization, bone formation |
| (Jiang et al., 2009) | Mandibular bone defect rat model | Fisher 344 rats  C= 18  T= 6 | Rat BM-MSCs  Pa: P2-P3  mSS implant and:  C= (-)/ MSC/ MSC-LacZ  T=MSC-BMP | Adenovirus/ BMP2, LacZ | wk-8-new bone formation, area, bone mineral density: T> MSC-LacZ= MSC> C(-)  residual scaffold: T= MSC-LacZ= MSC> C(-) | NR | BMP2 🡪Improve osteogeniesis |
| (Chang et al., 2003) | Maxillary defects pig model | Mitsae pig C= T= 20 | Pig auto BM-MSCs  Pa: NR  PLLA splint+ col implantation and:  C= MSC-βGal (left)  T= MSC-BMP (right) | Adenovirus/ hBMP2, βGal | New bone formation  T> C  Biomechanical property:  T= normal bone | NR | BMP2 🡪Improve osteogeniesis |
| (Fu et al., 2015) | Spinal L4-L5 postero-lateral defect rabbit model | New Zealand White rabbits  C= 12  T=6 | Rabbit allo BM-MSCs  Pa: NR  On site implantation-TCP and:  C= (-)/ MSC  T= MSC- BMP-VEGF | Baculovirus/ BMP2, VEGF | wk-12-bilateral spinal posterolateral fusion:  T> MSC> C(-) | No immune response to allo-MSCs or TCP | VEGF enhanced BMP2-induced bone healing by increasing vascularization🡪improve cell survival & bone homing |
| (Lin et al., 2011) | Massive bone defect rabbit model | New Zealand White rabbits  Bone volume- density, biomechanic, angiogenesis  C= NR+3  T= 40 | Allo -rabbit ASCs  Pa: P3-P5  On site implantation  C= MSC-Mock  T= MSC-BMP + MSC-VEGF-tr/ MSC-BMP + MSC-VEGF-per | Baculovirus/ BMP2, VEGF | wk-12-bone volume:  MSC-BMP+ MSC-VEGF-per> MSC-BMP+ MSC-VEGF-tr= NOS> C  wk-12- bone density:  NOS> MSC-BMP + MSC-VEGF-per > MSC-BMP + MSC-VEGF-tr > C  wk-12-biomechanical property: NOS= MSC-BMP + MSC-VEGF-per> MSC-BMP + MSC-VEGF-tr> C  wk-12-angiogeneis: MSC-BMP + MSC-VEGF-per> MSC-BMP + MSC-VEGF-tr= C | NR | Persisten BMP/VEGF expression 🡪improve bone healing |
| (Min et al., 2015) | Lung fibrosis (bleomycin induced)  mouse model | C57BL/6 mice  C= 4, T= 12 | Human UC-MSCs, Pa: NR,  C= saline-IV  T= ACE2 cells-IV/ UC-MSCs-IV/ UC-MSC-ACE2-IV | Lentiviral vector/ACE2 | d-7, d-14, d-28 – fibrosis score, collagen type 1 mRNA: UC-MSC-ACE2< UC-MSC= ACE2 cells< C | NR | ACE2 degrades Ang2 into Ang 1-7, Ang2🡪 promotes fibrosis, Ang 1-7 >< Ang2 |
| (Abdel-Mageed et al., 2009) | 9 Gy total body ɤ irradiation mouse model | BALB/c mice  C= 48,  T= 45 | BALB/c mouse MSCs, Pa: NR  C= d-1-PBS-IV  T= d-1-MSC-IV/ MSC-ECSOD-IV | Adenoviral vector/ ECSOD | d-35-survival: MSC-ECSOD= 52%, MSC= 9%, PBS= 10% | NR | scavenger of O2^-^ |
| (Xue et al., 2013) | Radiation induced lung injury mouse model | C57BL/6 mice  C= 10  T= 30 | C57BL/6 mouse BM-MSCs, Pa= P5  C= d-0 and d-14-PBS-IV  T= d-0 and d-14 AdV-sTGF-β-R-IV/ MSC-IV/ MSC-sTGF--β-R-IV | Adenoviral vector/ sTGF-β-R | 60 wk AI survival: C= 0%, AdV-sTGF-β-R = 20%, MSC= 40%, MSC-sTGF--β-R= 80%  d-120 lung fibrosis: C> AdV-sTGF-β-R> MSC> MSC-sTGF--β-R | 24 mo tumor formation: none | sTGF--β-R binds TGF—β 🡪 prevention of inflammatory cell infiltration, pro-inflammatory  cytokines, and collagen over production |
| (Zhang et al., 2014) | Radiation induced lung injury mouse model | C57BLmice  C= 18  T= 36 | Human UC-MSC, Pa: P3  C= d-1 AI – saline IV  T= d-1 AI – MSC/ MSC-CXCR4 | Lentiviral vector/ CXCR4 | d-7, d-30, d-60-AT:  CXCR4: C< MSC< MSC-CXCR4  SDF-1: C< MSC< MSC-CXCR4  TGFβ1: C> MSC> MSC-CXCR4  d-30-AT: Collagen type-1: C> MSC> MSC-CXCR4 | NR | CXCR4= receptor for stem cell SDF-1, CXCR4/SDF-1🡪 increase migration and homing capacity of MSCs |
| (Wang et al., 2015 a) | Radiation induced intestinal injury mouse model | C57BL/6 mice  C= 40  T= 80 | Human UC-MSCs, Pa= P3  C= h-6- saline-IV  T= h-6 MSC-IV/ MSC-HGF-IV | Adenoviral vector/ HGF | d-1, d-7, d-14, d-28 – AT:  EPT: MSC-HGF> MSC> C  AI: MSC-HGF< MSC< C  d-7, d-14, d-28 – AT:  IL-10: MSC-HGF> MSC> C  d-7- AT:  TNF-α, IFN-ɤ: MSC-HGF< MSC< C  d-14, d-28 - AT:  IFN-ɤ: MSC-HGF= MSC< C  d-14-AT: TNFα: MSC-HGF= MSC< C  d-28-AT: TNFα: MSC-HGF= MSC= C | Low HGF level🡪 no adverse event | HGF🡪 mitogen and anti-apoptosis of epithelial cells🡪 enhances intestine regeneration |
| (Zhang et al., 2019) | Radiation induced liver damage mouse model | SD rats  C= 15  T= 45 | SD rat AT-SC, Pa= P3  C= (-)  T= IIA-MSC-IV/ MSC-HGF-IV/ MSC-shHGF-IV | Lentiviral vector/ HGF, shHGF | d-2 AI: apoptosis, ALT, AST: C> MSC-shHGF> MSC> AT-SC-HGF  d-60 AI: collagen type-1: C> MSC-shHGF> MSC> MSC-HGF  d-60 AI: proliferation: C< MSC-shHGF< MSC< MSC-HGF | NR | HGF🡪 mitogen and anti-apoptosis of hepatocytes🡪 enhances liver regeneration |
| (Dey et al., 2010) | Huntington’s disease mouse model | YAC 128 transgenic  Mice  WTC= 12  C= 13  T= 52 | Mouse BM-MSC, Pa= P12  C= DMEM IS  T= MSC-BDNF-NGF-IS/ MSC-BDNF-IS/ MSC-NGF-IS/ MSC | Plasmid/ BDNF, NGF | Mo-4-mo-13: Rotarod 15 rpm LTF: WTC> MSC-BDNF> MSC-NGF> MSC-BDNF-NGF= MSC> C  Mo-4-Mo-13: Clasping: WTC< MSC-BDNF< MSC-NGF< MSC-BDNF-NGF= MSC< C  Mo-9: NeuN, DARPP-32 positive cells: WTC= MSC-BDNF> MSC-NGF= MSC-BDNF-NGF= MSC= C | NR | BDNF prevent neuronal loss  NGF- reduces striatal degeneration |
| (Liu et al., 2013) | Collagen induced rat and antibody induced mouse arthritis model | Fischer rat  T= 40  BalbC/SCID mice  NC= 10  T= 20 | Human BM-MSC, Pa: NR  Rat: T= BDO-Dex-IP/ Eta-IP/ MSC-IP/ MSC-hsTNFR-IP  Mouse: NC: (-), T= BDO-MSC-IM/ MSC-hsTNFR-IM | Retroviral vector/ hsTNFR | Rat: swelling ratio (right/left- NC):  d-0: MSC= Eta= MSC-hsTNFR= Dex  d-2, d-4, d-7: MSC> Eta= MSC-hsTNFR> Dex  Mouse: paw thickness:  d-3, d13, d17, d20: MSC> MSC-hsTNFR= NC  d-5, d-7, d-10: MSC> MSC-hsTNFR> NC | NR | MSC🡪 anti-inflammatory,  hsTNFR binds TNFα🡪 blocks TNF-α mediated inflammation |
| (Dong et al., 2020) | Rheumatoid arthritis (collagen induced) mouse model | DBA/1 mice  C= 10  T=20 | Human DP-SC, Pa= P3-P4  C= ADO-PBS-IV  T= ADO-MSC-IV/ MSC-HGF-IV | Adenoviral vector/ HGF | d-3, d-6, d-10 AT- arthritis (clinical): C= MSC-HGF= MSC  d-13, d-16, d-120 AT- arthritis (clinical): C> MSC-HGF= MSC  d-23 AT- arthritis (clinical): C> MSC-HGF> MSC  d-26 AT- arthritis (clinical): C= MSC-HGF> MSC  d-49 AT-synovitis (histological): C> MSC-HGF> MSC | NR | MSC🡪 anti inflammatory  HGF-dual effect: early phase🡪 immunosuppression effect, late phase 🡪 activate fibroblast-like synoviocytes🡪 IL6🡪 cell proliferation, decreased apoptosis |

LEW= Lewis rat (MHC haplotype: RT11) , BN= Brown Norway rat (MHC haplotype: RT1n), ACI= August Copenhagen Irish rat (MHC haplotype: RT1a), c= control, T= treatment, BM= Bone marrow, MSC= mesenchymal stem (stromal) cells, Pa=P= passage, PV= protal vein, Foxp3= transcription factor forkhead box P3, RAM= rat anti mouse, mAb= monoclonal antibody, d= day, RAI= Rejective Activity Index, PD-L1= programmed death ligand-1, ALT= alanine aminotransferase, AST= aspartate aminotransferase, TBIL= total bilirubin, DA= Dark Agouti rats (MHC haplotype: RT1n), min= minutes, RJV= right jugular vein, IL-= interleukin-,Th= T helper, IFN= interferon, TNF= tumor necrosis factor, TGF= transforming growth factor, MOG= myelin oligodendrocyte glycoprotein, SC= subcutaneous, IN= intra nasal, IP= intra peritoneal, CAR= chimeric antigen receptor, EAE= experimental autoimmune encephalomyelitis, allo= allogeneic, h= hu= human, NGF= nerve growth factor, SDF-1= stromal cell-derived factor-1, no= number, VEGF= vascular endothelial growth factor, HGF= hepatocyte growth factor, NSG mouse= NOD/SCIDIL2R?-/- mouse, B2M mouse= NOD/SCID ß2M null mouse, NS= Normosol= vehicle, Axt= axitinib= VEGF receptor inhibitor, wk= week, SD= Sprague-Dawley rats, Lcn2= Lipocalin 2, GFP= green fluorescent protein, AKI= acute kidney injury, nec= necrosis, Nr= normal rat, sCrea= serum creatinine, BUN= blood urea nitrogen, hPAMAM= hyperbranched polyamidoamine, LVEF= left ventricular ejection fraction, LVFS= left ventricular fractional shortening, LVAWTed= left ventricular anterior wall thickness at end-diastole, LVAWTes= left ventricular anterior wall thickness at end-sistole, LVIDed= left ventricular internal diameter at end-diastole, LVIDes= left ventricular internal diameter at end-sistole, CXCR4= C-X-C motif chemokine receptor 4, Dip= diprotin A, DPP-IV= CD26/dipeptidyl peptidase IV, LVDd= left ventricular end-diastolic dimensions, LVDs= left ventricular end-systolic dimensions, P= peritonium, Bcl-2= B-cell lymphoma 2 apoptosis regulator, +dP/dt= left ventricular maximum dP/dt, -(-dP/dt)= left ventricular minimum dP/dt, mo= month(s), C57BL/6-Tg (ACTbEGF)1Osb/J= GFP transgenic mice, Alg= alginate, auto= autologous, UP= ultrapure, RGD= arginine-glycine-aspartic acid, Col= collagen-1, ßgal= ß galactosidase, BMP2= bone morphogenetic protein, syn= syngeneic, allo= allogeneic, FK506= immunosuppresant, ASC= adipose derived stem cells, NOS= non operated site, tr= transient, per= persistent, MOI= multiplicity of infection, PLGA= poly(L-lactide-co-glycolide) scaffold, *= significant result, TCP= tricalcium phosphate scaffold, mSS= apatite coated silk scaffold, PLLA= bioresorbable polylactic acid plate. UC-MSCs= umbilical cord mesenchymal stem cells, NR= not reported, IV= intra-venous, ACE2= Angiotensin-converting enzyme 2, Ang= angiotensin, PBS= phosphate buffered saline, ECSOD= extracellular superoxide dismutase, O2^-^= superoxide anion, AdV= adenoviral vector, CXCR4= Chemokine (C-X-C motif) receptor 4, sTGF-β-R= soluble transforming growth factor-β type II receptor, wk= weeks, AI= after irradiation, h-= hour- , AT= after treatment, EPR= Epithelial proliferation rate, AI= apoptosis Index, IL= Interleukin, TNF= tumor necrosis factor, IFN= interferon, SD= Spraque- Dawley, AT-SC= adipose tissue derived stem cell, IIA= Immediately after irradiation, shHGF= small hairpin HGF that caused suppression of HGF, WTC= wild type control, DMEM= Dulbeco modified essential medium, IS= intra striatal, BDNF= brain-derived neurotrophic factor, mo-= month-, rpm= rotation per minute, LTF= latency to fall, NeuN= neuronal nuclear protein, DARPP-32= striatal GABAergic medium spiny neuron protein, NC= normal control, BDO= before disease onset, Dex= dexamethasone, Eta= Etanercept (hsTNFR)= human soluble tumor necrosis factor receptor type 2, DP-SC= dental pulp stem cell, ADO= after disease onset.
